# Wall Thinning Monitoring in Boiler U-Bends: A Review and Future Prospects with Fiber Optic Sensing

**DOI:** 10.3390/mi17050566

**Published:** 2026-05-01

**Authors:** Aayush Madan, Wenyu Jiang, Yixin Wang, Yaowen Yang, Jianzhong Hao, Perry Ping Shum

**Affiliations:** 1 School of Civil and Environmental Engineering, Nanyang Technological University, Singapore 639798, Singapore; aayush.madan@ntu.edu.sg (A.M.); cywyang@ntu.edu.sg (Y.Y.); 2 Institute for Infocomm Research, Agency for Science, Technology and Research, Singapore 138632, Singapore; jiang_wenyu@a-star.edu.sg (W.J.); wangyx@a-star.edu.sg (Y.W.); 3 Guangdong Key Laboratory of Integrated Optoelectronics IntelliSense, Southern University of Science and Technology, Shenzhen 518055, China

**Keywords:** boiler U-bends, wall thinning monitoring, fiber-optic sensing, FBG micromachining, non-destructive testing

## Abstract

Tube boilers are extensively employed in oil and gas refineries, as well as in petroleum, energy, and power generation industries, where they serve critical functions in local steam-generation units and combined-cycle gas turbine (CCGT) plants. However, these boilers are prone to defects arising from waterside corrosion (e.g., thinning of U-bend tubes), fireside corrosion, and material degradation caused by stress or creeping. Among these issues, wall thinning of tube bends is particularly severe, as it results in localized metal loss, reduced structural integrity, and an elevated risk of tube rupture or failure under high-temperature and high-pressure operating conditions. Such failures can significantly compromise boiler safety and efficiency, potentially leading to forced outages, costly unplanned repairs, or catastrophic damage if not detected in time. The current condition-monitoring policy for U-bends relies on scheduled preventive maintenance and unscheduled corrective interventions. In practice, this involves randomly checking approximately 10–20% of the tubes through spot scanning, partial scanning, or full scanning, with repairs typically carried out only after an undetected failure occurs. Such maintenance strategies generally require plant shutdowns, making the process time-consuming, labor-intensive, and ultimately not cost-effective. This paper reviews existing solutions, technologies, and research addressing the problem, and introduces femtosecond laser micromachined fiber optic sensors as a transformative approach for real-time monitoring of wall thickness reduction in U-bend boiler tubes, thereby opening pathways for further research.

## 1. Introduction

### 1.1. Background: Boiler U-Bends and Their Importance

Global energy demand is projected to increase by approximately 11–18% by 2050, with most of this growth attributed to developing regions. By mid-century, electricity is expected to emerge as the dominant energy carrier, driven primarily by the expansion of the transportation sector, data centers, and the large-scale adoption of green hydrogen technologies [1]. In the near term, global electricity demand is anticipated to rise at an annual rate of 3–4% through 2026, surpassing 29,000 TWh by that year [2].

Tube boilers are extensively employed in steam-generating facilities of the power and energy sectors, including local plants and combined cycle gas turbine (CCGT) systems. These boilers serve as a critical function by transforming thermal energy from fuel combustion or recovered exhaust gases into steam, which subsequently powers turbines to enhance overall energy efficiency and support reliable power generation. In response to growing energy demands, significant global advancements have been made in boiler operation, with particular emphasis on the improvement of U-bend boiler tubes’ operation, addressing performance efficiency, structural durability, and operational reliability. Boiler bends are specialized fittings installed within the piping network to facilitate fluid flow direction and accommodate mechanical stresses resulting from operational dynamics. As essential constituents of the pressure boundary in components such as boilers, superheaters, reheaters, and economizers, these bends are designed not only for geometric configuration but also for mechanical robustness under repetitive thermal loads and cycling. They let pipes change direction while accommodating thermal expansion and contraction in steam and hot water systems.

**Figure 1:**
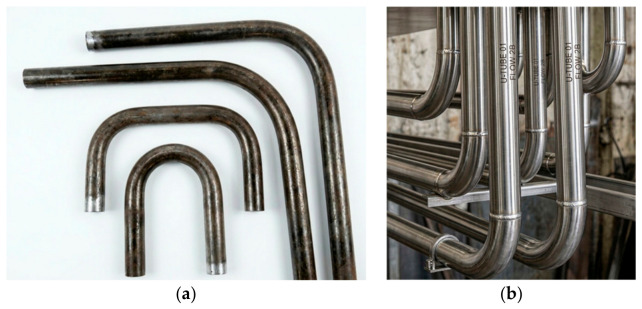
Illustration: (**a**) tube bend samples; (**b**) their arrangement in piping network. *(Generated using Gemini 3 Pro (Google) to illustrate shapes or conditions similar to those in [5,6]).*

Boiler bends come in a variety of radii and angles (e.g., 90°, 180°) and may be custom fabricated for superheater, reheater, and economizer elements within a boiler assembly, tailored for high-temperature, high-pressure environments. U-bends are specialized pipe sections shaped in a ‘U’ that redirect fluid flow (steam or water) and allow piping to return from one section of the boiler to another. They are commonly fabricated from carbon steel, stainless steel, or alloy steel to withstand high temperature, pressure, and corrosion and meet strict standards like AS 1228 [3] and EN 12952-5 [4] to ensure resistance to harsh conditions. A few examples of tube bends and their arrangement are shown below in Figure 1, including a U-bend, for illustration purposes only.

**Figure 2:**
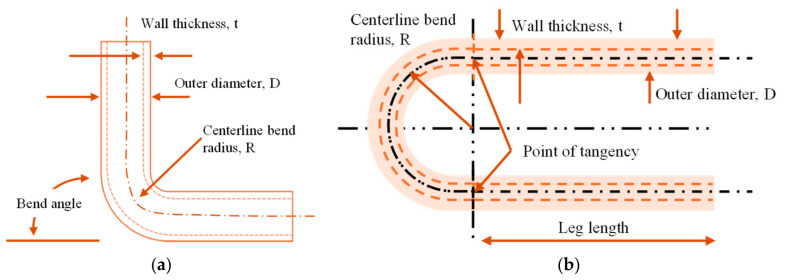
Schematic: (**a**) 90° tube bend; (**b**) 180° tube bend or U-bend.

U-bends are critical in steam and hot water pipes because they absorb the expansion and contraction resulting from constantly changing temperatures. Without U-bends, straight pipe runs would be subjected to excessive thermal stress, potentially leading to buckling, cracks, or failure. By allowing the pipe to flex, U-bends prevent stress from transmitting directly to pipe joints and supports. This increases the lifespan and reliability of boiler piping systems. Boiler U-bends are therefore essential for maintaining system integrity, safety, and operational efficiency in harsh environments. Typical schematics of a 90° tube bend and a 180° or U-bend are shown in Figure 2.

### 1.2. Wall Thinning in Boiler Tubes: Causes, Case Studies, and Implications

Facilities like boilers, pressure vessels, heat exchangers, condensers, etc. in any process plant require continuous monitoring for a long uninterrupted operation because any malfunction can cause complete operational shut down. Failures of boiler tubes are a leading cause of power plant shutdowns and have been frequently reported across many such facilities [7,8,9]. Degradation of components generally occurs due to process design, challenging surrounding conditions, design and manufacturing issues, etc. Due to prolonged exposure to harsh environments, tube bends are more vulnerable to structural defects, primarily by overheating [10,11], waterside corrosion, fireside corrosion, metal corrosion, vibrations, or a general failure of tube materials due to stress or creep [12].

**Figure 3:**
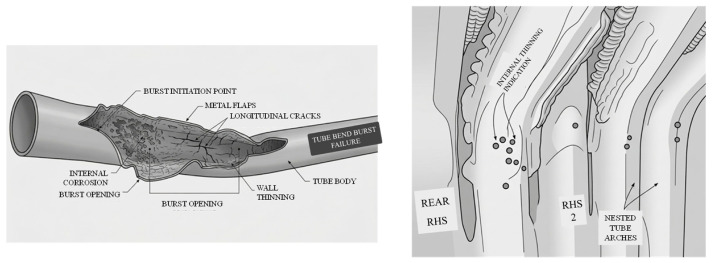

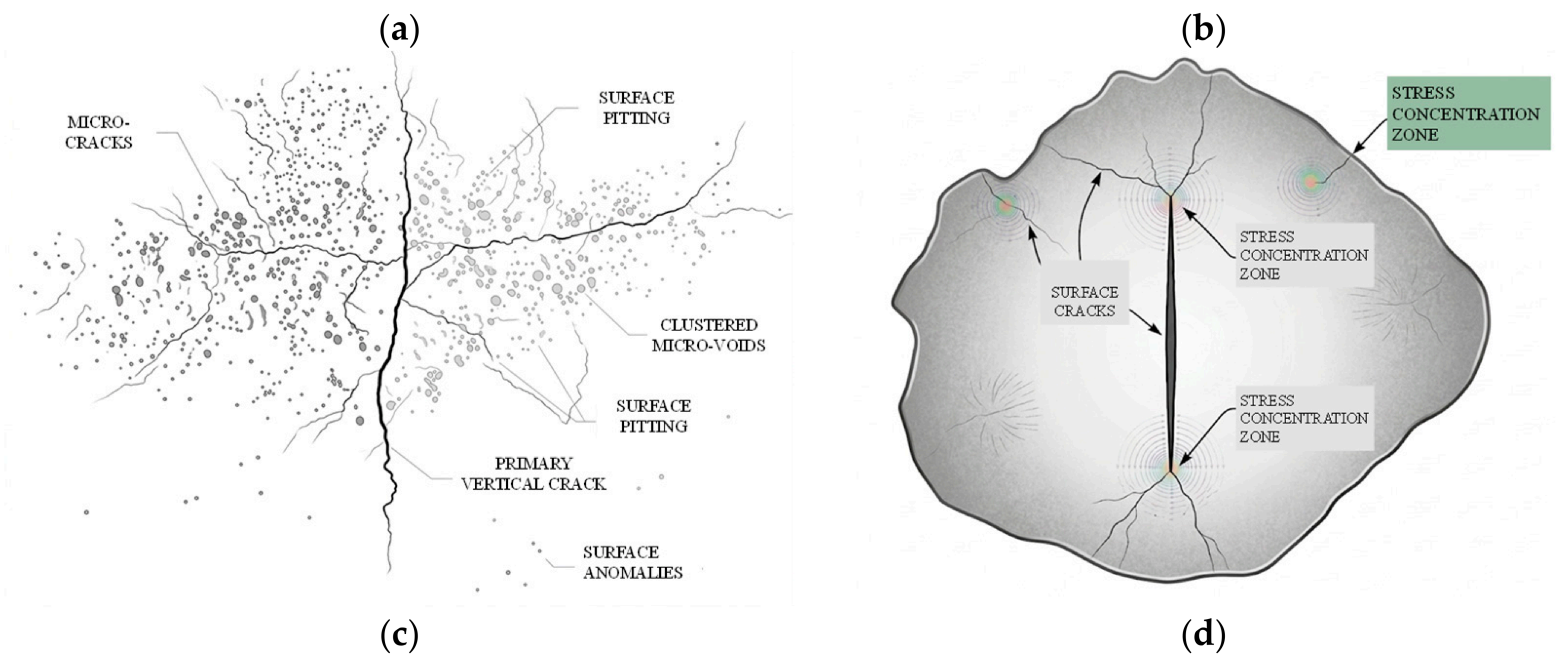
Tube bend failure case studies: (**a**) burst failure of water wall tubes; (**b**) varying wall thinning levels; (**c**) pitting and cracking in region of tube’s leakage; (**d**) cracks due to stress concentration. *(Generated using Gemini 3 Pro (Google) to illustrate shapes or conditions similar to those in [9,11]).*

External erosion from fly ash, blowing steam, or abrasive particles in flue gases can rapidly thin tube walls [13], especially at bends due to the change in flow direction. A few case studies have been reported studying the failure of water wall tubes, as shown in Figure 3, wherein a wide-open burst failure of the tubes is observed caused by non-uniform thickness reduction with respect to the walls (referring to Figure 3a, illustration only) and thinning levels in other tubes at the same level as the failed tube (referring to Figure 3b, illustration only). Tubes leaking due to overheating were investigated and found to experience pitting and cracking in the leaking region (referring to Figure 3c, illustration only), while stress concentration-induced cracks are reported in other cases (Figure 3d, illustration only). All these factors progressively cause uneven heat transfer, scale formation, removal of the oxide layer, and enhanced metal loss, which degrades a tube’s structural integrity and eventually leads to wall thickness reduction or the risk of rupturing. A case study [14] reported that high alkalinity (high pH water), oxygen pitting, and acidic dew point corrosion from sulfur compounds in low temperature flue gas led to localized thinning and material degradation at U-bends. Thinning exacerbates stress concentrations, and long-term overheating, creep (above 420–440 °C for carbon steel), and short-term thermal shocks can rapidly degrade tube integrity, sometimes resulting in “thin-lip” ruptures observed near bends [15].

A notable case from an industrial water tube boiler reported leakage and rupture in the bent sections of water wall tubes, as reported in [16]. Their investigations revealed that stress corrosion cracking (SCC) occurred due to tensile residual stresses combined with high dissolved oxygen in feed water. The cracked tubes showed localized thinning at U-bends, which became the failure initiation points. Another case study [17] showed cracking of the heat exchanger tube was primarily caused by SCC. A real-scale elbow specimen burst test was studied in [18], investigating how local wall thinning impacts stress distribution and failure pressure in curved tube bends, demonstrating that thinning significantly lowers structural integrity and can initiate fracture in these critical regions.

The authors in [19] studied the failure of a boiler water-wall tube where significant localized wall thinning on the fire-facing side was observed. The thinning was primarily caused by oxidation (fireside corrosion), which weakened the tube wall to below the pressure-bearing capacity. Microscopic and chemical analyses confirmed that oxidation and a reduced iron-to-oxygen ratio were responsible for this damage. Another case study [20] presented boiler tube failures due to fireside corrosion and embrittlement. It highlighted instances where pitting and sticking of fly ash containing corrosive elements (K, Ca) caused severe wall thinning, accelerating failure in reheater and superheater tubes. The study emphasized how increased temperature and fuel quality impact the progression of wall thinning and consequent tube failure. A comprehensive investigation in thermal power plants detailing the combined effects of corrosion, erosion, and overheating causing progressive wall thinning and weakening of tubes beyond tolerable limits is presented in [13]. The research introduced advanced ultrasonic measurement techniques for early detection of thinning and recommended methodologies for predicting remaining tube life considering thinning rates.

**Figure 4:**
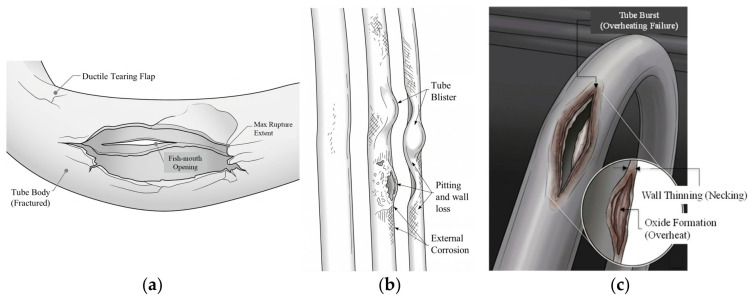
Tubes’ external appearance due to creep: (**a**) wide-open fish mouth fracture; (**b**) blistering; (**c**) failure due to short-term overheating. *(Generated using Gemini 3 Pro (Google) to illustrate shapes or conditions similar to those in [23,24]).*

Flow-accelerated corrosion (FAC) caused by phosphorus accumulation originating from water treatment agents, resulting in the removal of protective oxide layers and inducing thinning at the bend region below 1 mm, is reported in [21]. It is highlighted that with the increase in temperature, the corrosion rate rose and the yield strength of the tube declined. The wall thinning behavior of boiler tubes during the bending process is experimentally studied in [22], highlighting that excessive wall thinning might occur if bending technique and radius are inappropriate during the manufacturing process [22]. The external appearance of long-term overheating/creep-induced failing of tubes may range from small blisters to wide fractures [23,24], as shown in Figure 4 (illustrations only). Therefore, in such facilities, tube bends are particularly important due to their role in handling complex mechanical and thermal stresses in high-temperature, high-pressure environments.

Another major cause of defect is erosion [25] due to impurities present in heat or steam flowing within the tubes and flue gas molecules impinging on the surface with a high velocity, which may occur both internally and externally with respect to the tube. A change in the direction of flow will induce resistance to its flow at bends, which may continuously erode the tube, particularly with respect to water moving at an extreme velocity. Corrosion is random in nature, but with higher chances of occurrence at the tube bend. The wall thickness of tube bends directly affect their pressure, heat, and corrosion handling capacity due to the environment they are exposed to [26]. If the walls are too thin, they are prone to premature failure, i.e., bursting under high-pressure steam flow. On the contrary, if a wall is thicker, it might slow down heat conduction and reduce the heat transfer efficiency due to increased thermal resistance [27].

Hence, there is always a trade-off among safety, thermal efficiency, long-term reliability, and cost. These examples underscore that U-bend wall thinning is a significant risk factor for boiler tube failures. Its early detection and preventive maintenance are crucial to avoid large-scale failures. Hence, it is crucial to monitor thinning rates at U-bends, enabling operators to predict remaining tube life and intervene before ruptures occur.

## 2. Design Standards for Tube Bends and Wall Thinning Threshold

Tube design specification must adhere to international standards, notably ASTM (American Society for Testing and Materials), ASME (American Society of Mechanical Engineers), DIN (Deutsches Institut für Normung), and JIS (Japanese Industrial Standards), ensuring their quality and performance with variations in material and size tailored to specific needs.

The size of the tubes is generally defined by their outer diameter (OD) and wall thickness (WT), which varies depending on the required operating conditions. Typical considerations while selecting the WT include pressure handling ability, operation temperature, chemical composition, and mechanical properties [28,29,30,31,32]. Reference [26] outlines that the selection of the tubes’ wall thickness is often industry-specific. For example, power plants require relatively thicker tubes for usage in high-pressure steam systems, while oil and gas focus more on corrosion resistance with thickness adequate to handle aggressive chemicals. For a moderate temperature and pressure environment, carbon steel is sufficient, but alloying with chromium and molybdenum is essential to enhance creep resistance and strength in extreme conditions.

Design standards also standardize key tube design specifications, such as manufacturing (process: cold-drawn/hot-rolled; heat treatment: annealing/tempering/normalizing), mechanical properties (tensile strength/yield strength/elongation), chemical composition (thermal stability and corrosion resistance), etc. [32]. There are various national and international standards focusing on technical and design aspects of the tubes; however, this paper will concentrate on the following key standards:

- ASTM/ASME: A179 [33] (low-carbon steel); A209 [34] (carbon–molybdenum); A192 [35] (carbon steel); A210 [36] (medium-carbon steel); A213 [37] (ferritic and austenitic alloy); A335 [38] (ferritic alloy);
- DIN 17175 [39] (carbon/alloy steel tubes);
- JIS G3461 [40] (carbon steel) and 3462 [41] (alloy steel).

Table 1 below summarizes key parameters across specified standards for reference only. Pressure up to 9.8 MPa is considered medium, whereas conditions above 9.8 MPa are generally considered high pressure [32].

Table 2 provides a summary of the chemical composition or constituents under specified standards. The addition of chromium and molybdenum under A209/213/335 provides excellent mechanical strength, durability, and ability to withstand high pressure, temperature, and corrosive conditions, making it ideal for boiler applications.

Table 3 outlines a summary of mechanical properties under specified standards. Mechanical properties are critical to ensure the tubes can withstand operational conditions.

- Pressure resistance versus wall thickness:

It is clear from Table 1 and [28] that the pressure-bearing capacity of a tube bend largely depends on its thickness and outer diameter. Thicker tubes possess greater cross-section-resisting internal pressure, hence result in higher pressure capacity. Thermal conductivity and heat transfer are also significantly influenced by the tube’s wall thickness [42]. Since the wall behaves as a conductive barrier, a thicker wall increases thermal resistance and thereby lowers heat transfer efficiency. The heat flux q across the tube wall can be described by Fourier’s law of conduction, as below.

(1) $$q=k\frac{△T}{t}$$

where k is the thermal conductivity, △T is the temperature difference across the wall, and t is the wall thickness. As t increases, the heat flow decreases proportionally, lowering the overall heat transfer rate.

A primary determinant of a tube bend’s performance and outlining its figure-of-merit is the geometric ratio (GR), i.e., the ratio of a tube’s outer diameter to its wall thickness, also referred to as the D/t ratio, defined below [43,44,45,46].

(2) $$GR=\frac{Specified\;outer\;diameter\;of\;tube\;(D)}{wall\;thickenss\;of\;the\;tube\;(t)}$$

An article studying the pressure-bearing capacity of tubes/pipes for a range of D/t ratios is presented in [45]. It is observed that tubes with a lower D/t ratio tend to experience a higher collapse resistance and greater stability, allowing them to withstand higher pressure. Reference [43] highlights the tubes with a D/t above 20 (or t/D < 0.05) are prone to instability, ovality, or buckling. Another study [44] emphasized that a tube wall should not be thinner than 3% of its OD, i.e., t/D > 0.03.

An experimental study investigating buckling versus ovalization of steel tubes under bending loads concluded that buckling dominates in tubes with a D/t > 50 with negligible ovalization, whereas tubes with a D/t ranging from 16 to 50 experienced pronounced ovalization that weakens a tube’s stability [47]. Numerical simulations find that high D/t ratios lead to dynamic buckling propagation in long pipes, a concern with respect to U-bend safety margins [48]. Another study investigated the stability of circular tubes under symmetrical cyclic bending as influenced by the D/t ratio [49]. The raw tubes were externally machined to achieve the required D/t ratios while maintaining constant inner diameters.

The risk of wall thinning during the manufacturing process was examined by [50], outlining the importance of the bending radius and bending angle. The study concluded that at tighter bend angles, wall thickness risk grows with an increasing D/t ratio, meaning that tubes with a larger D/t ratio are difficult to manufacture at tighter bending angles.

For seamless tube bends operating at high temperatures, the D/t ratio generally falls within seven to 20 [46], with superheater tube bends typically positioned at the lower end of this range. A lower D/t (<7) provides more mechanical strength against collapse and tends to have higher creep resistance; therefore, such ratios are preferred in high temperature/pressure applications (superheaters). The standards do note that certain test cracks are acceptable and do not indicate rejection for tubes with a D/t ratio below 10 because excessive strain is introduced due to the tube’s geometry.

The JIS standards also emphasize that excessive D/t ratios can result in an increased risk of tube failure due to ovality, buckling, flattening, etc. Japanese standard G3452/59 specifies that tolerance on the outer diameter (with a tube’s original OD below 50 mm) is 1%; tolerance on wall thickness (original, t < 4 mm) is 12.5%; and thickness deviation (caused by thinning) is <20% [51,52]. A formula to calculate unit mass values for the tubes in accordance with Rule A of JIS Z 8401 [53] is provided below [52]. Unit mass reflects a way to quantify the amount of material present per length of tube bend, which is crucial in assessing how bending-induced wall thinning affects structural integrity.

(3) W=0.02466 t (D−t)

where W is the unit mass of a tube (kg/m) without including the socket, t represents wall thickness (mm), D is the tube’s outer diameter (mm), and 0.02466 is the conversion factor for obtaining W.

The DIN 2413 standard also specifies optimum tube material and dimensions targeting different applications, emphasizing wall thickness allowances during manufacturing [54]. DIN 2413-2:1993-10 specifically provides information pertaining to the design of bends and elbows for steel pressure pipes.

The following section presents widely used conventional non-destructive testing (NDT) techniques for regular monitoring of tubes’ wall thicknesses, or integrity at large, highlighting their respective advantages and limitations.

## 3. Conventional Monitoring Techniques and Motivation for Advanced Solutions

This section provides a summary of various NDT techniques suitable for integrity monitoring of tubes and pipes of different sizes, highlighting their underlying principles as well as their advantages and limitations. Current conventional monitoring techniques can primarily be categorized into the following categories [55,56]:

- Remote visual and digital video inspection (RVI or RVDI);
- Ultrasonic testing (UT);
- Radiography testing (RT);
- Eddy-current testing (ECT);
- Electromagnetic-induced acoustic wave transducer (EMAT);
- Infrared thermography (IRT).

### 3.1. Remote Visual and Digital Video Inspection (RVI or RVDI)

RVI/RVDI is a powerful technique for predictive or scheduled maintenance to monitor and check the health status of a plant’s assets. It uses visual techniques, i.e., video technology, capturing images from a distant location to allow an inspector to see the objects and materials from a distance. The inspection tools include borescopes, fiberscopes, push cameras, and crawlers. The advantage of this technique is remote operation, which is advantageous where direct human access is limited.

Typical applications for this NDT include process piping (oil and gas, pharmaceutical, and food preparation), turbines for power generation (steam and gas), aircraft engines (turbofan, turbojet, and shaft), aircraft fuselage, contaminated areas of nuclear power stations, and any areas which are generally directly inaccessible.

**Figure 5:**
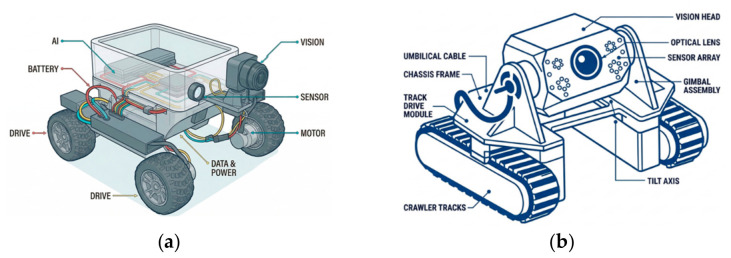
RVI examples: (**a**) turtle rover prototype; (**b**) MaggHD Crawler. *(Generated using Gemini 3 Pro (Google) to illustrate shapes or conditions similar to those in [58,59]).*

Study [57] shows the deploying of a robotic crawler or drones equipped with a camera assembly to navigate inside pipes, capturing close-up videos or images of internal surfaces. These systems may also integrate lighting and digital zoom to enhance observation of surface conditions like corrosion, cracks, or deposits without causing damage to the pipes; i.e., non-invasive systems. For illustration purposes, Figure 5 below shows advancements in remote sensing or measurement techniques and developed prototypes.

**Figure 6:**
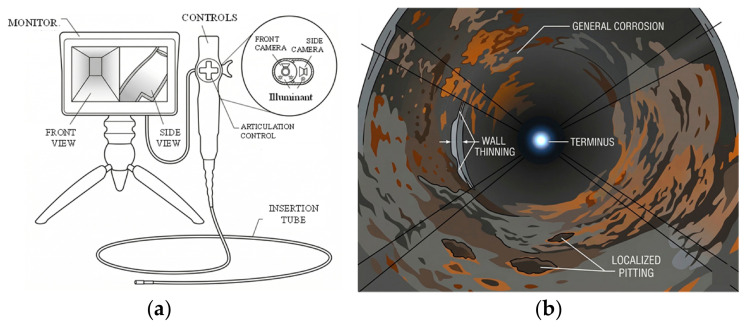

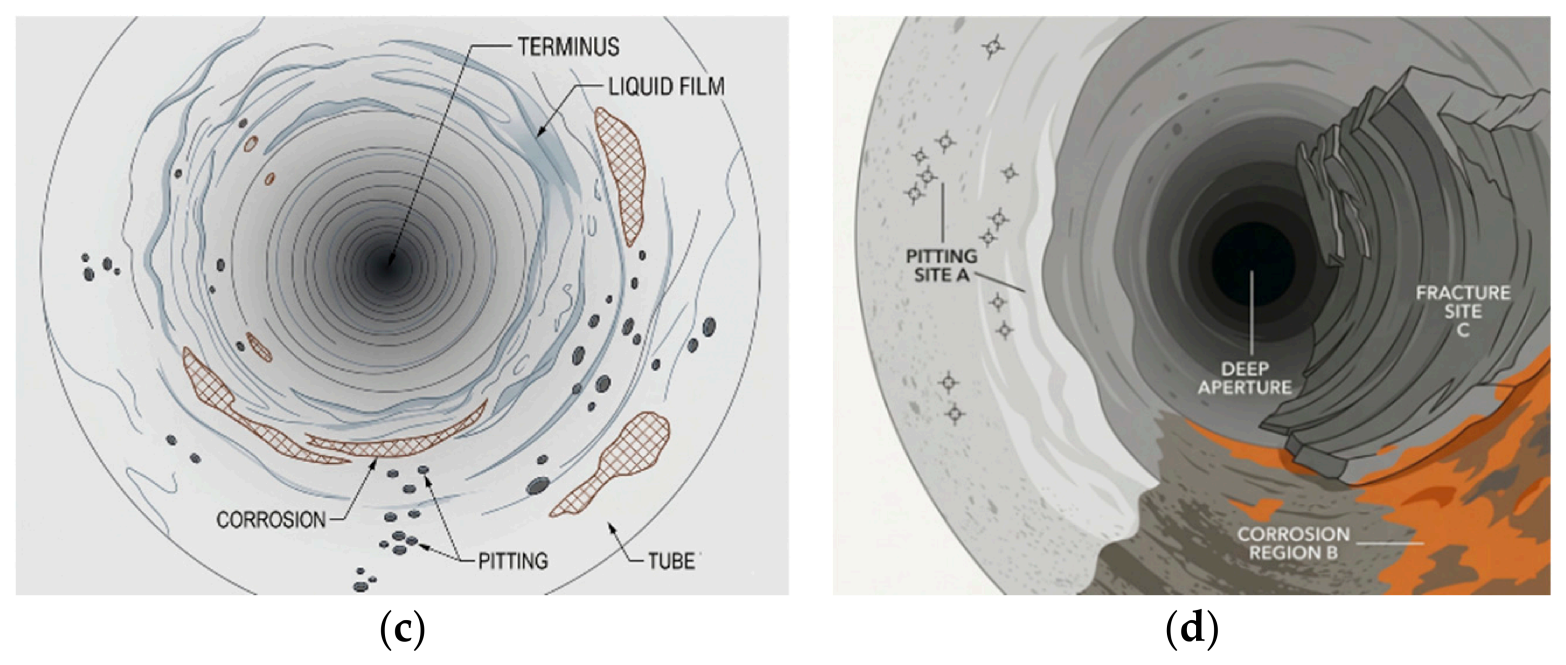
Modular videoscope for tube inspection: (**a**) inspection unit; (**b**) sample picture I; (**c**) sample picture II; (**d**) sample picture III. *(Generated using Gemini 3 Pro (Google) to illustrate shapes or conditions similar to those in [60]).*

Modular videoscopes are advancements in standard videoscopes with improved resolution, accuracy, and reachability, featuring design flexibility in which components like probe lengths, light sources, and control units are adjustable or allow customization. Modular videoscopes these days are more versatile, durable, cost-effective, and operationally flexible (360° camera orientation, night vision, etc.), offering improved data management, with, for instance, seamless transfer via Wi-Fi, USB, etc. Figure 6 below shows an example of a videoscope with extendable probe taking zoomed sample images of a tube’s inner surface.

- Advantages of RVI/RVDI techniques:
  ○ Enables inspection in confined spaces;
  ○ Significantly reduces inspection time and cost, as shown in a nuclear power plant seawater piping case study [57];
  ○ Can detect a broad range of defects, including pitting and fatigue cracks [61].
- Limitations of RVI/RVDI techniques:
  ○ Highly dependent on lighting conditions and cleanliness;
  ○ Reflective or curved surfaces challenge image quality [61];
  ○ Laser-based measurements are highly affected by the presence of water or other obstructions [58];
  ○ High initial cost of robotic system, licensing, and ownership [57].

Conventional RVI technologies face challenges operating in harsh environments like boilers or superheaters. Due to limited spatial coverage as scans are limited to outer surfaces only, RVI techniques are not efficient in detecting wall thinning of tube bends in such environments. Furthermore, the geometry of the elbow restricts the movement of inspecting probes, affecting image quality coverage. Hence, this approach is not the best solution to the problem stated. RVI execution requires skilled workers to operate devices like robots, drones, probes, etc. and interpret clues based on their experience, and is often perceived as necessitating plant shutdowns to operate effectively, causing increased downtime, at least for the use case mentioned.

Some notable advancements in RVI/RVDI include integration of computer vision algorithms [62] and LiDAR technologies, which allow precise measurements and defect quantification in inspection process [57,58]. Others propose inclusion of low-cost electronics like Raspberry Pi or Arduino, offering ease of control of the end device and transmitting of data wirelessly [58]. The adoption of AI-powered technologies with robotics offers features such as augmented and virtual reality, automation, etc., with deep learning enhanced video-based defect detection [63].

### 3.2. Ultrasonic Testing (UT)

**Figure 7:**
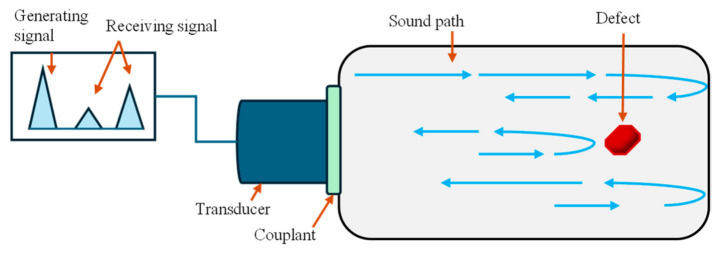
Principle of ultrasonic NDT approach.

Ultrasonic testing is a very standard NDT approach in which high-frequency sound waves are used to examine a test specimen, i.e., to detect internal flaws, measure thickness, and characterize materials. A conventional UT device operates on the piezoelectric principle, where a transducer generates high-frequency sound energy that propagates through the specimen. Reflections of these incident waves, either from internal flaws (if present) or from the specimen’s back surface, are captured and converted into voltage signals. The resulting spectrum indicates the flaw’s approximate location within the material considering arrival time of reflecting waves [64,65,66], as shown below in Figure 7. To ensure efficient transmission of sound waves into the specimen, a suitable coupling medium such as a gel is typically applied between the transducer and the test surface [67].

The frequency in UT scans lies in the range of 0.1 to 20 MHz (wavelength: 1 to 10 mm), while the velocity highly depends on the material, usually in the range of 1000–6000 m/s [68]. The resulting spectrum from UT scans can be visualized as Scans A, B, and C, which display energy vs. time (related to depth of the defect), a cross-sectional view, and a planner view of the flaw positions, respectively. UT executions are primarily conducted using handheld UT gauges, with full manual operations from skilled operators. More advanced ways of deploying UT testing in piping applications are as follows:

- Internal rotary inspection system (IRIS)

**Figure 8:**
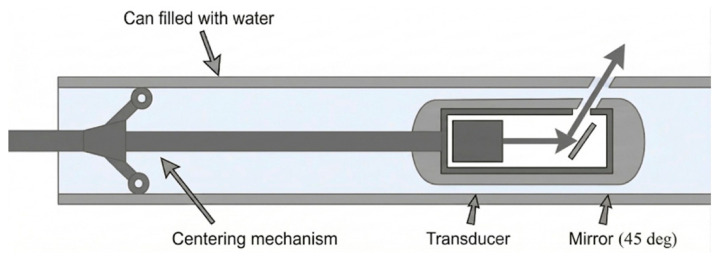
Principle of IRIS testing approach. *(Generated using Gemini 3 Pro (Google) to illustrate shapes or conditions similar to those in [71]).*

An IRIS (internal rotary inspection system) is an advanced version of UT mainly for tubular inspection [69], particularly in heat exchangers and boilers, to detect wall thinning, pitting, and corrosion. Its working principle, as illustrated below in Figure 8, is based on an immersion pulse-echo technique wherein the transducer is housed in a turbine head with a rotating 45º mirror which directs the signal radially into the tube wall [70]. The reflections from both the inner and outer layers follow the same path to the transducer, and the time interval between them is a measure of tube wall thickness. As the mirror rotates, the scan can sweep the entire circumference of the wall.

- Guided wave techniques (GWTs)

**Figure 9:**
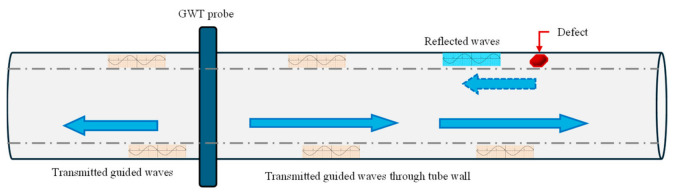
Principle of GWT testing approach.

The authors in [72] talk about regime shifting in UT from spot scanning (at the transducer’s position) to permanently installed monitoring systems via GWTs. GWTs involve propagation of ultrasonic waves along an elongated structure (e.g., pipes) for defect detection with minimal surface preparation. The working principle, summarized in Figure 9, involves attaching a ring of transducers around the circumference of the pipe to generate acoustic waves, which propagate along the pipe in both directions from the transducer’s position while ‘guided’ by the boundaries of the pipe wall (indicated by solid arrows in Figure 9).

Changes in the cross-section of the pipe, e.g., due to corrosion or erosion, thinning, etc., reflect the waves (indicated by dashed arrow in Figure 9). These defects can be located relative to the transducer’s position from the speed of the wave and arrival time of the reflected waves. The authors in [73] demonstrate low-frequency ultrasonic waves (5–250 kHz) propagating axially along pipes, followed by sensors detecting reflections caused by defects, allowing defect localization and size estimation.

Complementing GWTs with spot measurements (at selected locations) to accurately infer or validate information on wall thickness reduction is discussed in [72]. Wall thinning defect detection with dispersive circumferential guided waves around a pipe is proposed in [74]. Change in travel time of the guided waves around the pipes’ circumference is a measure of thickness reduction or the presence of a defect. Another study [75] highlights effective ways of applying Fourier and wavelet techniques for processing GWT signals under varying boundary conditions. Cylindrical guided waves are generated by piezoelectric transducers and made to propagate through the pipe wall boundaries.

Typically, standard UT sensors can only operate at a maximum temperature of 50 °C due to piezoelectric material depolarization and differential thermal expansion of different materials [76]; at elevated temperatures, special techniques or test methods must be employed, considering factors such as selection of coupling gel, velocity variation, gain adjustments, etc. UT-based conventional approaches for wall thinning detection at higher temperatures (150 °C) include a ‘Buffer-rod type UT’, i.e., inserting a buffer block between the transducer and the specimen, protecting the transducer at high temperatures, and a shear horizontal ultrasonic patch–catch waveguide system [77,78].

Study [79] presents an ultrasonic two-sensor thickness measurement technique that compensates automatically for temperature variations in real time by leveraging the different temperature dependencies of shear and compressive wave speeds. Mitsubishi developed a thin-film UT sensor for thickness measurements at elevated temperatures (up to 300 °C) [80]. Flexible metallic ultrasonic transducers are developed by depositing piezoelectric films onto titanium and stainless-steel membranes, capable of working at high temperatures (490 °C) [81]. the transducer can be installed on-site via appropriate glue or brazing onto steel pipes for continuous monitoring.

- Advantages of UT techniques:
  ○ Deep penetration capability;
  ○ Sensitive to subsurface defects and hidden discontinuities;
  ○ Applicable for a wide variety of materials, such as metals, composites, and ceramics.
- Limitations of UT techniques:
  ○ Require manual operation or essential training;
  ○ Less effective for porous materials;
  ○ Requirement of material-dependent coupling gel;
  ○ Issues at high temperatures due to difference in thermal expansion behavior between piezoelectric and coupling materials.

An in-depth study of UT techniques for flaw detection in construction and building materials is discussed in [82]. Research [83] highlighted circular ultrasonic arrays for an IRIS inspecting small diameter tubes and simulating acoustic beam profiles in the tube wall to select preferable parameters. A comparison of an IRIS with advanced magnetic flux leakage (MFL) techniques, as well as correlation with wall loss detection, is demonstrated in [84], highlighting its slow throughput. Applying an IRIS for nuclear power plant heat exchanger tube inspection, emphasizing its effectiveness and practical deployment, is explored in [85]. IRIS applications for steam generator and heat exchanger tube inspection are detailed in [86]. Modeling and simulation of IRIS and other UT techniques for tube inspection using software like CIVA are presented in [87].

Advancement in GWT techniques with inclusion of FPGA hardware enabling real-time damage identification and localization with respect to steel pipe inspection is presented in [88]. GWTs’ multimodal characteristics in pipes, i.e., longitudinal, torsional, flexural modes, enabling detection of axial and circumferential defects, are highlighted in [88,89]. Recent developments in UT outline advancements in signal processing, machine learning (e.g., transfer learning [90]), and digital twin [91] frameworks tailored for GWT-based structural health monitoring systems. A high-temperature ultrasonic guided wave transducer using lithium niobate piezoelectric ceramic is demonstrated in [92], claiming applications up to 600 °C.

Guided wave tomography [93,94]—the latest advancement in GWTs—employs two circumferentially spaced rings of transducers (one for transmitting guided waves, the other for receiving) to cover a pipe section, exploiting dispersion [95] where phase/group velocities depend on the frequency–thickness product. Full waveform inversion (FWI) iteratively reconstructs velocity maps by minimizing residuals between measured and synthetic waveforms, indirectly inferring thickness via dispersion curve interpolation. Velocity reconstruction often uses direct dispersion mapping and FWI handles anisotropic effects (e.g., in bends) via advanced modeling like Thomsen parameters. Another case study [93] demonstrates its efficacy in inspecting a 220 mm diameter pipe bend, featuring a 100 mm diameter defect, with particularly strong performance for defects near the extrados position. However, this approach has limited applicability to boiler tube bends, which typically involve much smaller diameters (~50 mm), closely spaced tubes, and internal wall thinning at the intrados due to erosive flows. These geometric and defect characteristics hinder direct translation to robust, commercial end-to-end monitoring solutions in harsh environments.

### 3.3. Radiography Testing (RT)

**Figure 10:**
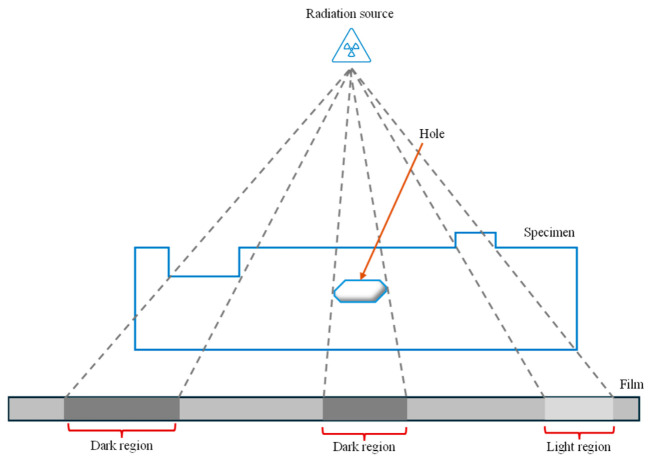
Principle of RT testing approach.

RT is a versatile NDT technique that uses ionizing radiation such as X-rays or gamma rays to penetrate in the test piece and examine the volume of the material without causing any damage [96]. The working principle can be understood from Figure 10. The test specimen is placed between the radiation source and detector on which the traces are produced. The produced images may contain information revealing cracks, voids, porosity, etc., which is significantly valuable for monitoring structural integrity. Safety protocol and licensing are essential while performing RT because the work involves usage of ionizing radiation.

RT can be further subcategorized as follows:

- Film radiography: using photographic films to capture images in a darkroom;
- Computed radiography: creating digital images on phosphor imaging plates scanned by lasers;
- Digital radiography: using digital detector arrays for real-time image generation and instant viewing;
- Computed tomography: producing 3D cross-sectional images through multiple angles’ pixels.
- Advantages of RT techniques:
  ○ Ability to detect internal and sub-surface flaws that are not visible from outside;
  ○ Non-contact and non-destructive;
  ○ Rapid scans, reduced inspection time, and quick decision making.
- Limitations of RT techniques:
  ○ Hazardous to operate;
  ○ Highly directional and sensitive to flaw orientation;
  ○ Depth and exact location of discontinuity are not provided;
  ○ Limited portability due to radiation source.

In boiler applications, radiography with digital detector arrays like CMOS and phosphor plates are prominently used. A prototype digital radiographic system developed by EPRI includes a 300 kV X-ray source, a segmented CMOS detector, robotic positioning inside the boiler, and software for image acquisition and alignment. It is effective for detecting cracking and thinning through multiple layers, including refractory and insulation inside boilers [97,98]. Mitsubishi has developed a technology based on digital radiography, evaluating crack depth evaluation through image density profile analysis in detecting a variety of cracks [99]. Computed radiography with phosphor plates provides clear images detecting corrosion fatigue cracking and weld quality [100]. Recent advancements in RT include advanced imaging methods like terahertz and infrared thermography, which complement radiography by providing non-contact and safer inspection options [101]. Complementing RT with other NDT techniques to improve defect detection and material characterization is presented in [102,103], highlighting that one NDT technique operating solely may not suffice for complex evaluations.

### 3.4. Eddy Current Testing (ECT)

**Figure 11:**
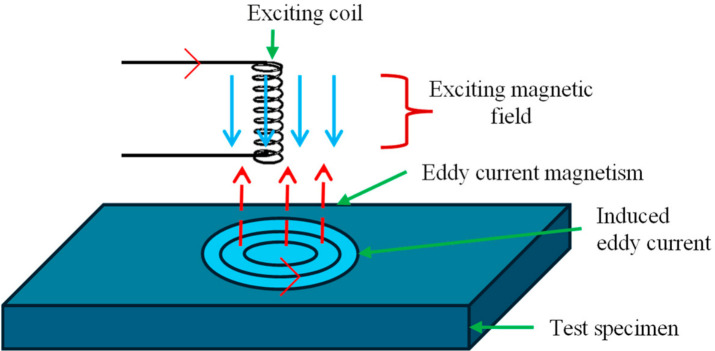
Principle of ECT testing approach.

The basic principles of ECT rely on electromagnetic induction. An alternating current applied to a coil generates a varying magnetic field around the coil. When this coil is brought near a conductive material, it induces circulating electrical currents called eddy currents in the material. These eddy currents create their own secondary magnetic field that opposes the primary field generated by the coil, according to Lenz’s Law. Any flaws or discontinuities in the conductive material, such as cracks, corrosion, or changes in thickness, disrupt the flow and strength of the eddy currents. This disruption alters the secondary magnetic field, which is detected by the probe and analyzed to identify the presence and characteristics of defects [104,105]. The eddy current density is highest near the surface, making this method sensitive primarily to surface and near-surface defects. The working principle can be well understood from Figure 11.

Testing methods include eddy current array (array of electrically charged coils), Lorentz force ECT (using multiple DC magnets overcoming skin effect), and surface array ECT in aerospace applications [106].

- Advantages of ECT techniques:
  ○ Highly efficient and sensitive in terms of the detection of near-surface defects or imperfections;
  ○ Capable of measuring electrical conductivity and coating thickness;
  ○ Non-contact and non-destructive, applicable at high temperature;
  ○ Fast and efficient.
- Limitations of ECT techniques:
  ○ Only applicable for conductive materials, i.e., not applicable for plastics, ceramics, composites, etc.;
  ○ Eddy current penetration depends on a material’s conductivity, magnetic permeability, frequency of alternating current;
  ○ Effective for near-surface defects due to shallow penetration depth.

Advance usage of ECT in thickness evaluation of thermally sprayed coating exposed to boiler tube environments based on impedance spectrum of the measurement coil is reported in [107]. Article [108] emphasizes combining ECT with RVI, i.e., robotic crawlers with electromagnetic techniques for remotely inspecting a boiler’s wall tubes. Pulsed ECT enables larger depth penetration and improved coverage. An excessive eddy current array, with multiplexing of multiple coils allowing for a large-area scan in a single iteration, is detailed in [109].

ATF industrial solutions introduced an AI-powered solution, e.g., the “Eddyvisor S Ai”, combining multi-frequency eddy current signals with AI models to predict critical parameters with high precision [110]. Reference [111] reports further AI-driven analytics platforms that perform real-time flaw detection and classification. Systems like “Eddyfi Technologies’ Magnifi software” utilize AI models to validate signal data quality and automatically highlight critical flaw indications during tube inspections.

### 3.5. Electromagnetic-Induced Acoustic Wave Transducer (EMAT)

**Figure 12:**
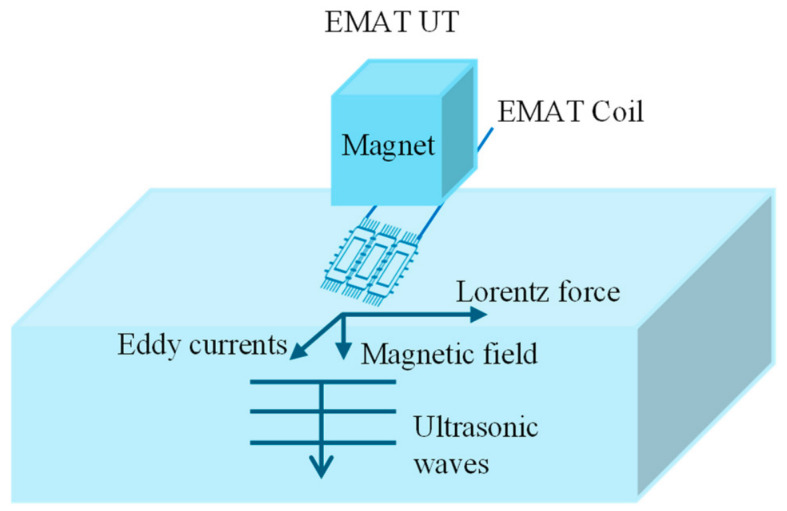
Principle of EMAT testing approach.

An EMAT can be understood as a kind of ultrasonic testing technique without needing the coupling agent. The basic diagram is shown below in Figure 12. An EMAT is a non-contact inspection approach wherein the ultrasonic waves are generated in the material using electromagnetism without physical contact. Ultrasonic waves are induced using two interacting magnetic fields, i.e., first via a high-frequency alternating current field from a coil and secondly via a static magnetic field via magnets [112,113]. These fields induce eddy currents in the surface of the material, which experience Lorentz forces that produce ultrasonic waves within the material lattice. The same electromagnetic principles allow an EMAT to receive ultrasonic echoes by detecting the waves interacting with the magnetic field, inducing currents in the receiving coil.

- Advantages of EMAT techniques:
  ○ Portable, lightweight systems, allowing volumetric inspections;
  ○ Applicable in harsh environments, i.e., remote, high temperature, hazardous;
  ○ Non-contact approach, eliminating the need for coupling gels.
- Limitations of EMAT techniques:
  ○ Lower sensitivity and expensive compared to UT probes;
  ○ Applicable to conductive and ferromagnetic materials;
  ○ Limited penetration depth, not suitable for deep flaws.

An advanced EMAT can generate various wave modes and polarizations, such as longitudinal, shear (vertical and horizontal), Rayleigh, Lamb, etc. [114], which are challenging to produce with standard piezoelectric UT transducers. An EMAT is well suitable in high temperatures or environments with limited access. The biggest challenge with EMATs is that the generated signal is weaker than piezoelectric UT transducers, requiring strong magnetic fields or rare-earth magnets, such as samarium–cobalt (SmCo) and neodymium–iron–boron (NdFeB) or pulsed electromagnets, to achieve strong magnetic strength, temperature stability, and corrosion and demagnetization resistance. NdFeB magnets are among the strongest commercially available permanent magnets, generating powerful magnetic fields that enhance EMAT signal strength and sensitivity. SmCo magnets are slightly less strong but still far superior to conventional magnets, enabling more efficient eddy current generation and Lorentz force [115]. Advanced EMAT techniques are outlined below.

- Hybrid LASER-EMAT

**Figure 13:**
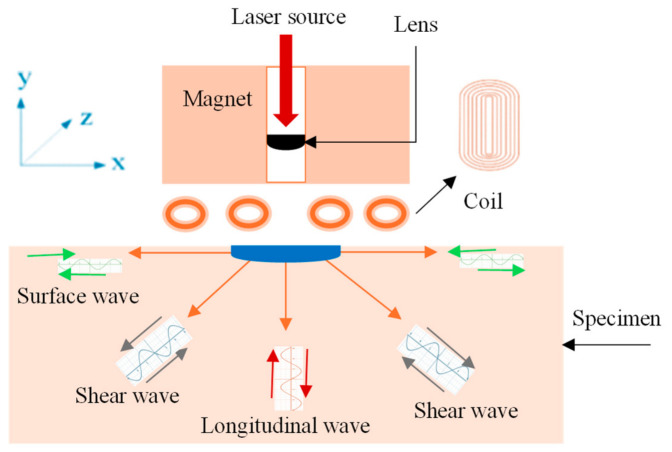
Principle of hybrid LASER-EMAT testing approach.

EMATs combined with lasers is a hybrid approach to enhance sensitivity of standard EMAT transducers; i.e., high-energy laser pulses are illuminated at the specimen’s surface to generate ultrasonic waves of various modes that are detected with an EMAT transducer, revealing defect detection, material properties, or thickness monitoring [116]. Interference among various arrival signals results in a poor signal-to-noise ratio, causing complicated signal demodulation. The operating principle is shown below in Figure 13 [117], wherein a laser is focused via a lens and excited ultrasonic waves in the specimen being tested. A voltage signal is induced across the coil in the presence of a static magnetic field generated by the magnet–signal reception. The article in [117] presents a numerical study using a hybrid approach with respect to simultaneous measurement of the width and depth of metal plates.

Optimizing design parameters of the hybrid LASER-EMAT approach—for example, magnet height, EMAT coil radius, operability at elevated temperatures (300 °C)—are studied in [118]. It further presents the effect of specimen temperature on the received ultrasonic signal’s amplitude. A LASER-EMAT ultrasonic shear wave resonance method for detecting damage in high-temperature alloys, offering a large lift-off between the sensor and material, is presented in study [119]. The method utilizes multi-feature fusion in amplitude–frequency domain imaging for rapid, high-resolution detection. Inducing ultrasonic waves by thermoelastic and surface constraint mechanisms are studied in [120]. It further proposes longitudinal wave resonance method for plate thickness gauging at high temperatures. Rayleigh waves generated from a laser are converted into shear waves that propagate through the specimen [121].

- Millimeter wave (mmWave)

The mmWave technique involves the application of electromagnetic waves in the frequency range of 30–300 GHz (wavelengths: 1–10 mm) to inspect materials. It measures changes in permittivity, signal reflection, or transmission to reveal defects, thickness loss, or other anomalies, especially in dielectric and composite materials. The mmWave approaches are more sensitive to electromagnetic properties and do not rely on acoustic signals [122]. Signals with short wavelengths can capture tiny anomalies as well [123].

Another study [124] investigated Synthetic Aperture Radar (SAR) imaging with frequency modulated continuous wave radar to build high-resolution 2D images of internal structural features or defects. The study also highlighted the importance of range migration algorithms (RMAs) and compressed sensing (CS) in SAR imaging methods in enhancing the signal-to-noise ratio and resolution of the produced images. The authors in [123] carried out a detailed review of mmWave non-destructive testing and evaluation, mentioning that specialized antennas (microstrip patch, dipole) and probes can be embedded or scanned over a surface to detect dielectric changes, cracks, corrosion, or disbands. The usage of mmWave techniques in detecting defects such as voids, disbands, and delamination inside low-loss dielectric composites and foam materials is detailed in [125]. Signals in the microwave to mmWave bands can penetrate thick insulation materials and reveal internal anomalies in real-time with portable devices. Compared to other NDT methods, mmWave techniques are less common in boiler-related applications due to (i) high operating temperature environments; (ii) the complex geometries of tubes; and (iii) the limited penetration depth. However, the technique is summarized above to aid in the reader’s understanding.

### 3.6. Infrared Thermography (IRT)

**Figure 14:**
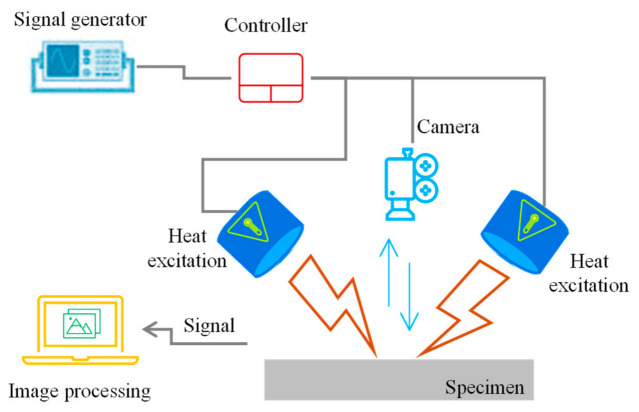
Principle of IRT testing approach.

Infrared thermography is an NDT technique that detects subsurface defects or anomalies in materials by monitoring surface temperature variations using an infrared camera, as shown below in Figure 14. It works on the principle that all objects above absolute zero emit infrared radiation, and differences in thermal properties or defects cause distinct temperature contrasts appearing on the surface [126].

It is either implemented by detecting a natural temperature difference in materials (passive approach) or involves external excitation (via flash lamp, heat guns, etc.) inducing thermal contrast. The active approach includes techniques like pulsed thermography, pulsed phase thermography, and lock-in thermography, enabling fast detection of surface defects. Thermal waves generated by heating or cooling propagate through the material, and defects affect heat conduction, causing surface temperature deviations detectable by the IR camera [127]. The technique is typically effective only for near-surface defects due to limited penetration depth. Additional coatings are often required at the test surface to enhance their emissivity. In ultrasonic infrared thermography [128,129], ultrasonic energy is exploited to generate heat at defects, improving contrast by localized friction heating but requiring pressure application that may risk secondary damage.

- Advantages of IRT techniques:
  ○ Fast and rapid inspection, easy-to-interpret images;
  ○ Non-contact and non-invasive;
  ○ Large coverage area.
- Limitations of IRT techniques:
  ○ Only limited to surface temperature detection;
  ○ Challenges to operate on reflective surfaces;
  ○ Emissivity dependence;
  ○ Skilled operators needed to read thermograms.

There has been significant research investigating IRT techniques in boiler tube inspections, as follows. Development of a thermal imaging system consisting of a moving thermal line source and IR imager to quantitatively measure wall thinning due to corrosion on steel boiler tubing is presented in [130]. The study emphasizes theoretical modeling and dynamic calibration to relate temperature changes to material loss (or thickness indirectly). Reconstructed images of corrosion-induced material loss are obtained from measurements of surface temperature variations and subsequently analyzed. A similar approach using a line heat source and IR imaging for corrosion-induced thinning detection is investigated in [131]. EPRI’s report provides applications of IRT techniques to detect air leakages, insulation deterioration, and temperature abnormalities in boiler components [132]. A thermal imaging approach to analyze temperature distribution and cleaning effectiveness on boiler tube walls is presented and discussed in [133]. Applications of IRT methods for defect detection and temperature condition assessment of process heater tubes are explored and presented in [134].

### 3.7. Summarizing Key Attributes and Operational Constraints of Existing NDT Techniques

In this subsection, Table 4 synthesizes and critically compares existing NDT techniques based on their key advantages, limitations, typical sensitivity (e.g., SNR), and penetration depth, wherever applicable. Table 5 further summarizes their applicability to high-temperature environments and complex tube bend geometries, as well as their capability for quantitative versus qualitative wall thickness monitoring.

Subsequently, Table 6 below presents a comparative summary of recent case studies on wall thickness reduction detection, emphasizing their respective performance metrics like resolution (understood as accuracy/error), sensitivity (or SNR improvement), penetration depth (max measurable thickness or range), speed (real-time meas. capability), and robustness (to surrounding environment), wherever possible. Limited direct comparisons exist across papers, as tests use specific pipes (study-specific), but we believe it will help in understanding trends.

Most of the existing NDT methods fall short for real-time, online tube bend condition monitoring with predictive analytics due to manual scanning or offline access, making them impractical for continuous deployment in hard-to-reach locations where accessibility is limited by operational constraints. Guided wave approaches suffer from high signal attenuation around curvatures, poor resolution for distributed strain mapping, and inability to perform predictive modeling without periodic interruptions. Even advanced options like acoustic emission or eddy current testing lack the multiplexing capacity for quasi-distributed sensing and struggle with data volumes needed for machine learning-based prognostics in harsh environments.

Next, we provide Table 7, which highlights the key shortcomings of conventional NDT methods and demonstrates how fiber optic sensing addresses them through permanent installation, discrete or distributed measurement capabilities, high-temperature resilience, and multiplexing for online predictive assessment. Additionally, we acknowledge that research specifically targeting tube bend wall thickness monitoring using fiber optic sensors under boiler operational conditions remains largely unaddressed. In subsequent sections, we explore how fiber optic sensing is transforming the oil and gas industries through diverse applications, followed by a forward-looking approach that highlights its potential for wall thickness monitoring of tube bends.

## 4. Fiber Optic Micromachined Sensors: Transforming Boiler Tube Condition Monitoring

Initially developed in early 1960s [150], fiber optic technologies have transformed the modern world by enabling high-speed communication networks that underpin global data transfer and cloud computing, forming the backbone of data centers, internet infrastructure and much more. In sensing applications, optical properties of the light signal, e.g., intensity, wavelength, phase, polarization, etc., are altered to measure a measurand. Over the last decade, optical fiber technology has transformed industrial sensing, empowering advanced solutions in fields like structural health monitoring, energy systems, and manufacturing by providing precise and distributed measurements that were previously unattainable with conventional sensors. Fiber optic sensors enable real-time, high-precision monitoring of parameters such as temperature, strain, pressure, and vibration. Their immunity to electromagnetic interference, high sensitivity, versatility, and ability to operate in harsh and remote environments make them superior to conventional electronic sensors for many applications.

The technology supports distributed sensing along long distances, allowing spatial and temporal multiplexed measurements on a single fiber (limited by a finite spatial resolution), point sensing focusing on localized distinct positions for monitoring, or quasi-distributed (multiplexing enabled multi-point) sensing.

### 4.1. Distributed Fiber Optic Sensing (DFOS): Summary Aiding Broader Context

**Figure 15:**
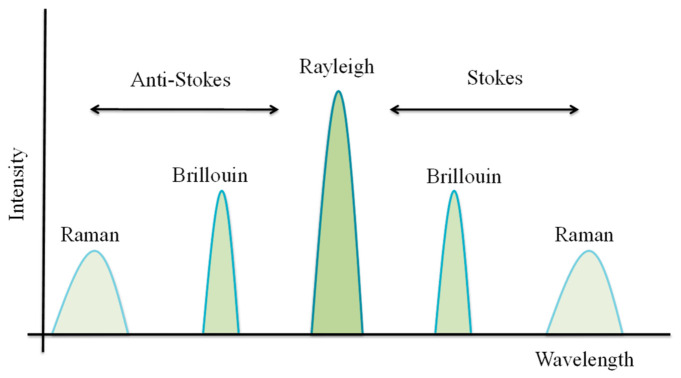
Backscattering in optical fiber (adapted from [151]).

This section outlines a summary of DFOS and related applications in oil and gas industries, providing readers with a broader context. DFOS leverages the backscattering of the light signal traveling within the fiber, with particularly effects such as Rayleigh, Brillouin, and Raman, as shown in Figure 15 below. Rayleigh-based sensing technologies include optical time-domain reflectometry (OTDR), distributed acoustic sensing (DAS), optical frequency-domain reflectometry (OFDR), and optical backscattered reflectometry (OBR) [151]. Brillouin scattering is used in other sensing schemes, such as Brillouin optical frequency domain analysis (BOFDA) [152], the Brillouin optical frequency domain reflectometer (BOFDR) [153], Brillouin optical time domain analysis (BOTDA) [154], and the Brillouin optical-time domain reflectometer (BOTDR) [155], offering a range of spatial resolution, accuracy, and distance coverage capabilities. Raman scattering is primarily limited to temperature measurements [156].

**Figure 16:**
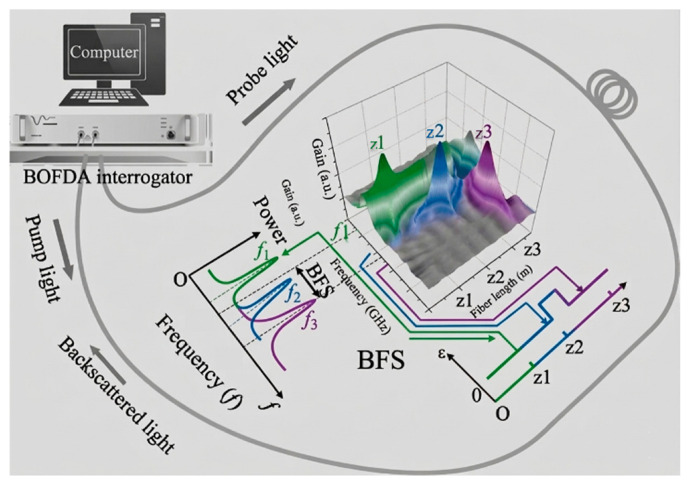
Illustration: Brillouin optical frequency domain analysis, or BOFDA. *(Generated using Gemini 3 Pro (Google) to illustrate shapes or conditions similar to those in [160]).*

This method allows continuous sampling of data from a long length of optical fiber, limited by a finite spatial resolution (for example ≤ 1–2 m). DFOS finds applications in real-time monitoring of large structures, e.g., railway tracks, tunnels, bridges, pipelines, dams, etc., offering higher sensitivity and sampling rates [157,158,159]. The authors in [160] beautifully present usages of both distributed fiber optic and point sensing in monitoring performance of a tunnel liner. Figure 16 below explains the working of a Brillouin optical frequency domain analysis (BOFDA) that monitors the Brillouin-frequency-shift (BFS) of the optical fiber that carries information with respect to strain and temperature change.

**Figure 17:**
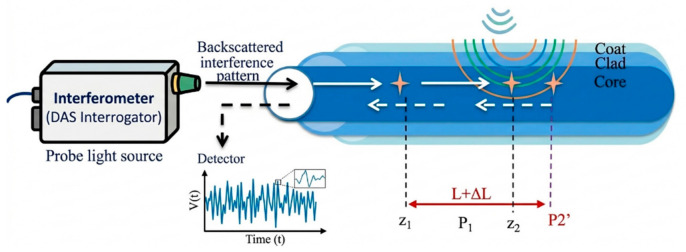
Illustration: Distributed acoustic sensing, or DAS. (*Generated using Gemini 3 Pro (Google) to illustrate shapes or conditions similar to those in [151]).*

In DAS, acoustic events occurring on the fiber generate compression or elongation altering the phase or amplitude of the scattered light within the optical fiber. The interrogator continuously sends optical pulse signals (at a fixed rate) and monitors the back-reflections, as shown below in Figure 17. Study [151] demonstrates deployment of optical fiber DAS in railway track monitoring, advancement in railway defects detection with machine learning [161], and graphing of neural networks combining data fusion with an accelerometer [162]. The high sampling rate of optical fiber sensing systems enables the collection of vast amounts of real-time field data, which machine learning algorithms can efficiently analyze for accurate event detection.

**Figure 18:**
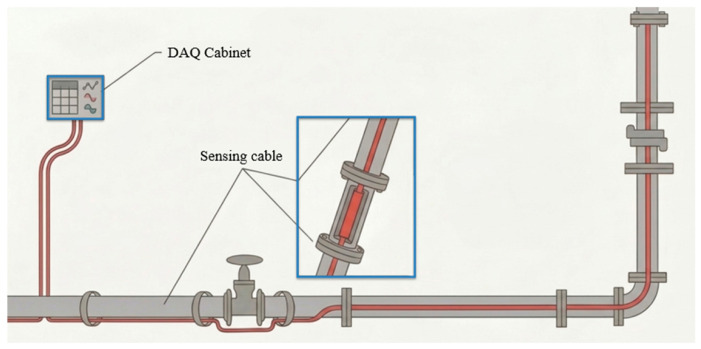
DFOS-based pipeline leakage detection system. *(Generated using Gemini 3 Pro (Google) to illustrate shapes or conditions similar to those in [166]).*

Returning our focus to energy industries, DFOS is widely used in pipeline monitoring for leakage detection, corrosion, structural deformation, third-party interference, and seismic activities in real time over long distances. Practical applications of DFOS in pipeline monitoring highlighting its economic benefits and prevention of pipeline thefts are discussed by Bandweaver [163]. DFOS-enabled continuous monitoring of pipelines with respect to leak detection and localization in different leak scenarios is demonstrated in [164]. Deformation and integrity monitoring of pipelines using fiber optic sensing textiles is presented in [165]. The effectiveness of DFOS in leak detection of pressurized liquefied gas LNG and ammonia pipelines is explored in [166], which can be understood from the illustration shown in Figure 18.

**Figure 19:**
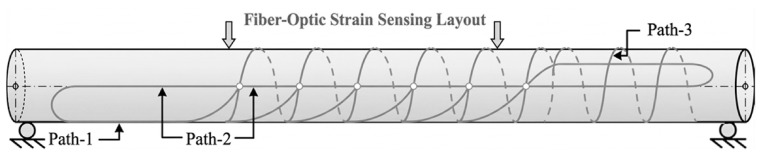
Distributed fiber optic strain sensing layout. *(Generated using Gemini 3 Pro (Google) to illustrate shapes or conditions similar to those in [167]).*

Monitoring of interactive bending and deformation using a distributed fiber optic strain sensing layout arranged along different paths, enabling detection, localization, visualization, and quantification of interactive defects, is demonstrated in [167], and illustrated below in Figure 19 for more understanding. The cabling layout across various paths can be observed, enabling wholistic monitoring of a pipeline.

**Figure 20:**
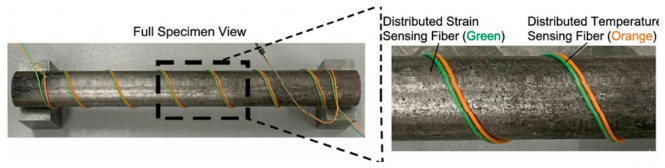
Illustration: DFOS deployment on a pipe specimen. *(Generated using Gemini 3 Pro (Google) to illustrate shapes or conditions similar to those in [171]).*

**Figure 21:**
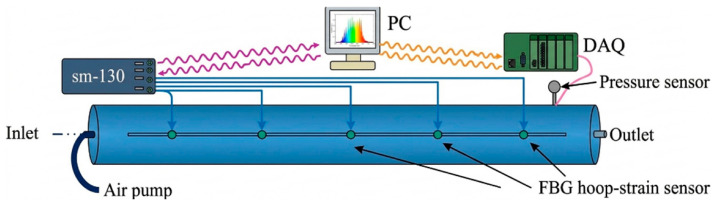
Diagram: DFOS-based measurement system. *(Generated using Gemini 3 Pro (Google) to illustrate shapes or conditions similar to those in [172]).*

DFOS based on an OTDR approach for flow monitoring in natural gas pipelines is presented in [168]. Modeling and experimental verification of pipeline leakage using fiber optic distributed temperature sensing is studied and disclosed in [169]. Experimental exploration of pipe–soil interactions using DFOS is studied in [170]. Distributed strain sensing for pipeline corrosion monitoring and its quantification using analytical approaches is presented in [171], as well as corrosion monitoring via fiber optic hoop strain detection [172], as illustrated below in Figure 20 and Figure 21, respectively.

**Figure 22:**
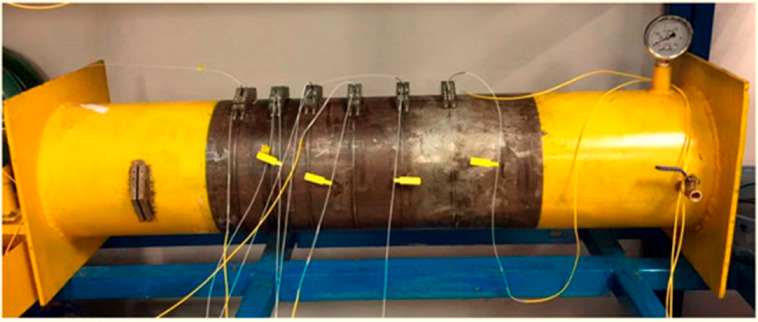
Fiber optic sensing pipeline model [177].

DFOS-enabled helix strain measurements in steel bars for corrosion monitoring are explored in [173], and corrosion quantification via mass-loss of steel piles is proposed in [174]. A recent work [175] demonstrates the capability of fiber optic DAS in detecting illegal intrusion of underground pipelines, enhancing its application range in theft monitoring. Similarly, flow and blockage detection in slurry pipelines using fiber optic DAS is explored in [176]. Hoop strain monitoring using fiber optic point sensors addressing risk of leakage and corrosion in pipelines is proposed and experimentally simulated in [177], as shown below in Figure 22. Results demonstrated that variations in hoop strain consistently reflected pressure changes and wall thinning, validating the sensor’s capability to detect both leakage and corrosion.

Table 8 below summarizes case studies of fiber optic sensors applied in oil/gas/pipeline monitoring for improved readability, including underlying technologies, target applications, evaluation environments (lab test or field trial), and performance parameters such as sensitivity, error, etc., wherever applicable.

### 4.2. Fiber Bragg Grating (FBG) Sensors

**Figure 23:**
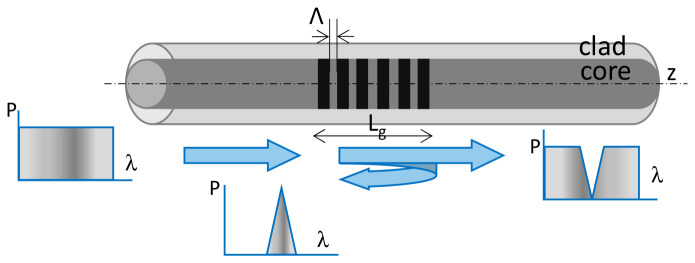
Schematic: FBG structure in an optical fiber.

FBG sensors are realized as grating structures within the core of an optical fiber, referring to a periodic perturbation of the refractive index in the core, as shown below in Figure 23.

Such a grating structure in the fiber resonates and reflects a specific wavelength of light, following the Bragg criteria given by Equation (4):

(4) $$\lambda_{B}=2{\;\eta }_{eff}\Lambda$$

where,${\;\eta }_{eff}$ represents the effective refractive index of the fiber core, Λ denotes the grating period or pitch, and $\lambda_{B}$ is the Bragg wavelength or reflection wavelength. Variations in strain and temperature applied to the grating modify its effective refractive index and/or period, leading to a shift in the Bragg wavelength, as described by Equation (4). Therefore, the FBG structure functions as an intrinsic sensor for measuring strain and temperature at the grating location.

Equation (5) below, derived from (4), shows that a shift in Bragg wavelength ($∆\lambda_{B}$) carries information related to strain (ε) and temperature (T) changes. The first term corresponds to strain-induced response, wherein $P_{e}$ represents the photo-elastic coefficient. The second term corresponds to a shift due to temperature changes considering the thermal expansion coefficient ($\alpha_{\Lambda })$ and thermos-optic coefficient ($\alpha_{\eta }$).

(5) $$\frac{∆\lambda_{B}}{\lambda_{B}}=\left(1-P_{e}\right)\cdot ∆\varepsilon +\left(\alpha_{\Lambda }+\alpha_{\eta }\right)\cdot ∆T$$

These gratings are usually inscribed by exposing the fiber core to UV lasers and are classified as Type I or Type II, depending on their photosensitivity and response to UV irradiation. Key methods to fabricate FBGs include the phase mask technique, point-by-point writing, and interferometric approach, as summarized in [192]. Apodised gratings feature a tapered UV-laser intensity profile along the grating, which suppresses side-lobes in the reflection spectrum and thereby enhances multiplexing capabilities, making them advantageous for strain and temperature monitoring applications [193]. Let us now turn directly to FBG sensor packaging and examine how micromachined FBGs are well-suited for monitoring wall thinning of boiler tube bends.

- FBG Packaging: A Key Determinant of Performance

Packaging of the FBG sensors is the primary determinant of their performance, robustness, and longevity. Without proper packaging, FBGs may remain fragile or fail to operate effectively for a given application. Proper packaging shields FBG sensors from moisture, chemicals, and mechanical wear, safeguarding against degradation and performance drift. Well-designed packaging ensures ease of installation, repeatability of measurements, and protection during handling and operation, overall extending sensor service life. Sensor packaging also carries special consideration as it impacts strain-transfer characteristics, temperature cross-sensitivity, form-factor and stability. Hence, the role of FBG packaging is crucial for its reliable functioning. Some trends are summarized as follows.

Carbon fiber-based packaging for FBG embedment measuring strains on wind turbine blades is presented in [194]. A wearable FBG sensor featuring cross-shape packaging and a sinusoidal configuration for displacement-based strain monitoring is disclosed in [195]. A novel polydimethylsiloxane packaging with a gecko-inspired microarray structure has been proposed for sensor protection and strain isolation for more accurate temperature measurement [196]. A comprehensive review discussing FBG packaging solutions, such as hermetic sealing, metal or polymer coatings, additive manufacturing, etc., for environmental and biochemical applications, and highlighting challenges like thermal mismatch and packaging-induced strain, etc., is provided in [192].

**Figure 24:**
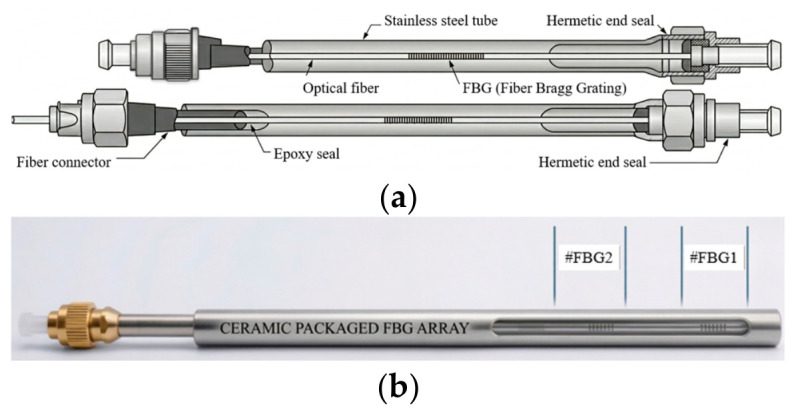
Fire-resistant FBG packaging by OFCN: (**a**) seamless steel tube; (**b**) ceramic packaged FBG array. *(Generated using Gemini 3 Pro (Google) to illustrate shapes or conditions similar to those in [197,198]).*

Fire-resistant packaging of FBG sensors using single or multi-layers of seamless steel tube capillaries (all metal) is offered by OFCN [197], illustrated below in Figure 24. Such packaging is highly desirable for non-combustible FBG sensors working in high-temperature environments. An array of FBG sensors is also demonstrated with ceramic packaging enabling high-temperature multipoint sensing probes or FBG thermometers [198].

**Figure 25:**
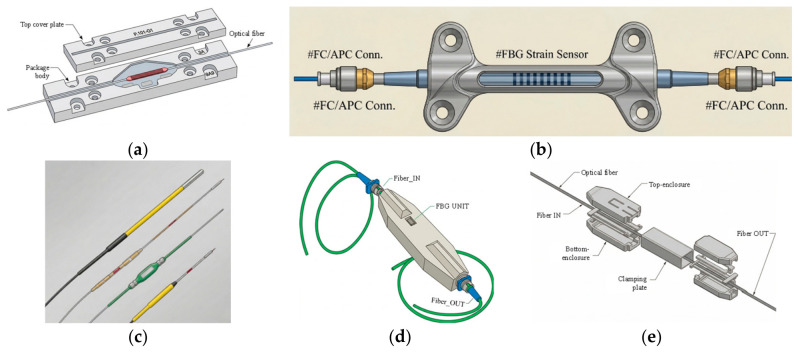
Various FBG packaging available: (**a**) from AFR; (**b**) from ATGRATING; (**c**) from Micronor Sensors; (**d**) from Redondo Optics; (**e**) from EON Photonics. *(Generated using Gemini 3 Pro (Google) to illustrate shapes or conditions similar to those in [199,200,201,202,203]).*

FBG packaging for high-power fiber lasers compatible with kilowatts of pump power is provided by AFR [199]. Surface-mounted FBG strain sensor packaging using dual clips suitable for installation at concrete or steel surfaces is offered by ATGRATING [200]. Various kinds of FBG sensing probes from Micronor Sensors [201] enable multipoint quasi-distributed sensing of temperature and strain. Customized FBG packaging suitable for various applications such as shocks, cracks, acoustic emissions, etc., is offered by Redondo Optics [202]. EON Photonics proposed a glass or carbon-fiber based composite packaging of FBG sensors primarily for avionic applications [203]. All these packaging solutions are depicted in Figure 25.

- Harnessing FBGs’ Sensing Capability in Oil and Gas

Diverse applications in the oil and gas industries, such as fluid level, temperature, strain and vibration, pressure, gas leakage monitoring, etc., have been explored using FBG sensors. These sensors are primarily preferred due to their tolerance to harsh environments, immunity to electromagnetic interference, and multiplexing capability along pipelines.

A fiber optic liquid level sensor based on Archimedes’ principle, using a suspended mass attached to an FBG strain sensor, is introduced in [178]. As the liquid level rises, buoyant force reduces the load on the FBG, hence decreasing strain. The sensor’s performance largely depends on mass geometry and material through strain transfer characteristics. FBG sensing in wastewater monitoring, measuring chlorine and lead contaminants, is proposed in [179]. The sensor exploits wavelength shifts with changes in refractive index characteristics for such chemical detection. FBG-embedded diaphragms with a temperature compensation scheme for liquid level measurement is demonstrated in [180]. Samples were tested in an industrial water tank, where the system achieved a ninefold improvement in thermal stability, high linearity, superior sensitivity (2.8 pm/mm), and significantly lower temperature error (1.04 mm/°C) compared to recent diaphragm-based sensors. Integration of FBG within a double-flange cylinder filled with flexible polymer polydimethylsiloxane (PDMS), realizing a liquid pressure sensor, is presented in [181]. The flexible design allows the sensor’s measuring range and sensitivity to be tuned by modifying the structure, enabling high performance across different liquid levels.

FBG-based water level sensor using a PVC cap filled with silicone rubber, where two FBGs were embedded for strain and temperature monitoring, is disclosed in [182]. A 2 m float generates compressive stress on the rubber under buoyancy, inducing measurable strain in the fiber. FBG samples were tested at depths of 5 mm, 10 mm, and 15 mm, yielding sensitivities of 155.7, 80.7, and 43.5 pm/m, corresponding to maximum measurable water heights of 5 m, 10 m, and 18 m, respectively. A theoretical model studying silicon rubber depth and water level affecting the sensor’s response was also presented. FBG-based temperature and pressure sensors packaged with solid glass particles for downhole monitoring are studied in [183]. An FBG sensor network for petroleum hydrocarbon leak detection via strain monitoring is investigated in [184]. An FBG sensor array for temperature monitoring along vacuum chambers of magnets in a synchrotron radiation source under a harsh environment (radiation plus strong magnetic fields) is studied in [185].

Custom-designed FBG for acetylene gas detection using optical cross-correlation technique is introduced in [186]. Unlike conventional absorption-based methods, this approach offers higher sensitivity and signal-to-noise ratio while reducing cost and complexity. By fabricating aperiodic Bragg gratings that replicate the absorption spectrum of the target gas, the system enables precise and robust detection of gas concentrations. Experimental results confirm that the method can discriminate between varying concentrations of acetylene while remaining resilient to interference from other gases, demonstrating the potential of customized FBGs as compact, low-cost, and tunable photonic devices in such applications.

FBG sensors for CO_2_ gas detection while remaining immune to temperature and humidity are proposed in [187], using a polysulfone/polyimide (PSF/PI) film with polyethylene glycol (PEG) as surfactant. The PSF/PI/PEG composite with hydrophobic and superhydrophobic coatings prevents water interference. The authors in [204] highlight FBG-based hydrogen sensors for industrial hydrogen detection, emphasizing safety, accuracy, and rapid response. They emphasize hydrogen-sensitive materials (like Palladium, WO3 and constituting metal catalysts dopants), coatings, and advances in sensor structure and methods to improve FBGs’ durability. The sensing mechanism relies upon variations in strain or temperature caused by the chemical interaction between the hydrogen and sensitive materials. The capability of FBG to detect the moisture content of sandstones while eliminating the impact of temperature and humidity by advancing the packaging with moisture sensitive dual-gel composite materials is developed in [188].

Article [189] highlights the suitability of FBG sensors for precise measurement of longitudinal strain in boiler hanger rods to enable real-time force and load change monitoring. Multiple sensors on a single fiber are efficient for multi-point non-invasive stress monitoring under varying load conditions. Development of an FBG sensing system integrated with an optical band pass filter and photodetector to monitor real-time temperature changes in structures under dynamic thermal loading, such as steel beams, suitable for thermal cycling environments in typical power plants is demonstrated in [190]. Fabrication of heat-resistant FBG sensors and their embedment in metal molds (e.g., silver brazing or metal adhesives) for monitoring high-temperature pipelines and components such as sodium cooling pipelines, maintaining performance at temperatures up to 500 °C relevant for industrial power plants, is presented in [205]. Mitsubishi [191] proposed multipoint temperature measurement using optical fiber sensors (including FBGs) to produce useful data for operation and periodic inspection of thermal power plant boilers, contributing to better thermal management. FBG modeling and analysis to improve temperature monitoring and design optimization for industrial applications is provided in [206].

FBGs have been effectively demonstrated for pipeline intrusion and theft detection by detecting strain, pressure, or vibration change via peak reflection wavelength. For example, a non-intrusive, high-sensitivity FBG structure for pipeline pressure and intrusion detection has been experimentally validated with high strain sensitivity [207]. The sensing principle is developed by analyzing pipeline wall stress under internal pressure, and the design incorporates a flexure hinge with a mechanical lever to enhance sensitivity. Patented cable-based FBG sensors for perimeter intrusion detection [208] show deployment in protective cables around fences, detecting vibration and strain signals specific to intrusion events.

- Strategy for wall thinning monitoring using FBG sensors

Under constant conditions, such as temperature, internal gas flow, pressure, etc., if the corrosion of the tube bends occurs, resulting in a change in inner wall thickness, its strain on the outer wall will certainly change accordingly. Having the FBG sensor surface mounted on the outer wall of the tube, when other conditions are constant, enables the FBG sensor to directly measure and quantify the strain changes at the outer wall. During long-term monitoring, if no thinning is induced, the measurement of strain and temperature of a so-called good-condition tube will show consistent reading or trend. When the thinning occurs, the strain reading will change slowly and ultimately push the readings into abnormal zones, where alarms can be triggered. Hence, it is important to have an analytical understanding of various parameters. Let us now delve more into constructing a strategy, as follows.

Hoop stress $\left(\sigma_{h}\right)$ is defined with respect to rotationally symmetric objects, such as tubes acting circumferentially and perpendicular to the axis, and with respect to the radius of the cylinder wall. These stresses are primarily generated due to the pressure gradient. Hoop stress produced by an internal pressure (P) on a pipe with radius (r) is inversely proportional to its thickness (t) [209], given by Equation (6), Barlow’s formula, as follows, For a given pressure and pipe radius, increasing the wall thickness reduces the hoop stress, and conversely for thinner wall thickness.

(6) $$\sigma_{h}=\left(\frac{P\cdot r}{t}\right)$$

**Figure 26:**
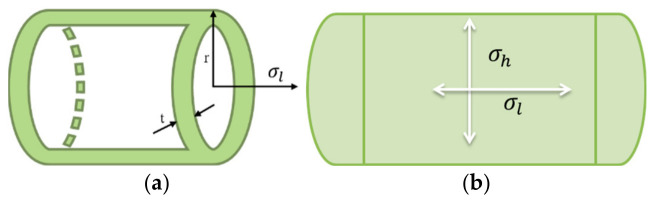
Illustrations of hoop stress: (**a**) illustration I; (**b**) illustration II (adapted from [209,210]).

For a closed cylinder (tube), the internal pressure also creates a force along a tube’s axis, producing longitudinal stress, $\sigma_{l}$, given by Equation (7), following [210]. Hoop stresses for thin-walled tubes are generally twice the longitudinal stresses, resisting the tubes’ circumferential bursting. Both hoop and longitudinal stresses on a tube can be understood by Figure 26.

(7) $$\sigma_{l}=\left(\frac{P\cdot r}{2t}\right)$$

For thick-walled pipes, the stresses vary along the thickness, such as the maximum stress occurring at the inner wall surface compared to outer surface, leading to more complex formulations, which are out of the scope of this paper. Typically, thin-walled tubes have a thickness-to-inner diameter ratio ≤ 0.05 [211]. The study shows that under a maximum pressure of 7 MPa, hoop stress reaches approximately 200 MPa, while the longitudinal stress is in the range of 100 MPa.

Hoop stresses at a tube bend or elbow may differ from a straight section and may not be straightforward. The impact of the curvature effect must be accounted for when evaluating the stress behavior in the bend region. Significance of the curvature effect largely depends on the ratio of the bending radius (BR) to the pipe diameter (D). Measuring hoop stress-induced strains on tube bends with a given Dt ratio under varying internal pressure may help in understanding thickness versus strain behavior, and eventually the tube’s failure or threshold limit with respect to bursting stresses. Studying stresses in orthogonal directions in combination with internal pressure and the Dt ratio for thin-walled tubes with fiber optic sensors (mainly the FBGs) at the elbow or bend location could be a next-generation inspection approach for energy industries, enabling real-time monitoring without human intervention. The monitoring can further be automated to run on frequent intervals without shutting down the plants, hence saving revenue which would have otherwise been lost with manual approaches.

- Analytical assessment relating strain levels with wall thickness (or thinning) and internal pressure

Here, we present a quantitative assessment through an analytical discussion that correlates strain levels with wall thickness and internal pressure, thereby complementing the conceptual framework outlined in the subsequent section.

**Figure 27:**
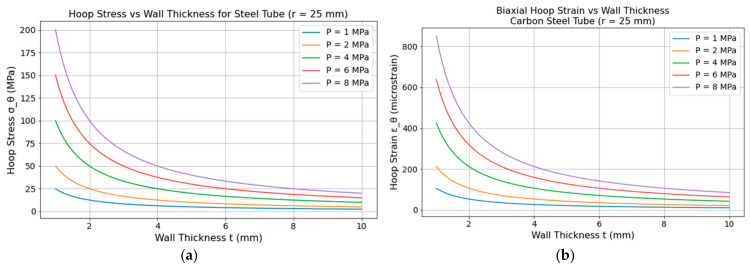
Analytical study at fixed pressure and varying wall thickness: (**a**) hoop stress; (**b**) biaxial hoop strain.

Equation (6) above shows that for a fixed pressure and tube radius, increasing the wall thickness reduces the hoop stress ($\sigma_{h}$), and conversely for thinner wall thickness. Considering a tube with an outer diameter of 50 mm, the following figure, Figure 27a, shows the pressure-induced hoop stress with respect to wall thickness for internal pressures ranging from 1 MPa to 8 MPa. It follows an inverse relationship, i.e., stress decreases hyperbolically as thickness increases. At fixed pressure, hoop stress drops sharply at low thickness, i.e., a reduction from 3.0 mm to 2.8 mm already produces a measurable increase due to the steep slope in the thin wall regime. Higher pressures shift all curves upward proportionally, amplifying stress risks.

Subsequently, we extend this to examine biaxial hoop strain behavior by incorporating the Poisson effect and longitudinal stress ($\sigma_{l}$), as described in Equation (9), where ν and E denote Poisson’s ratio and Young’s modulus, respectively. Using Equations (6) and (7) above, biaxial hoop stress and strain equations can be written as:

(8) $$Biaxial\;hoop\;stress=\;{(\sigma }_{h}-\nu \cdot \sigma_{l})\;\;=\left(1-\frac{\nu }{2}\right)\cdot \sigma_{h}$$

(9) $$Biaxial\;hoop\;strain=\frac{\left(1-\frac{\nu }{2}\right)\cdot \sigma_{h}}{E}$$

For carbon steel tubes, Young’s modulus is typically around 200 GPa and Poisson’s ratio is approximately 0.3. Figure 27b below shows that hoop strain (induced) also follows a similar hyperbolic trend with wall thickness.

**Figure 28:**
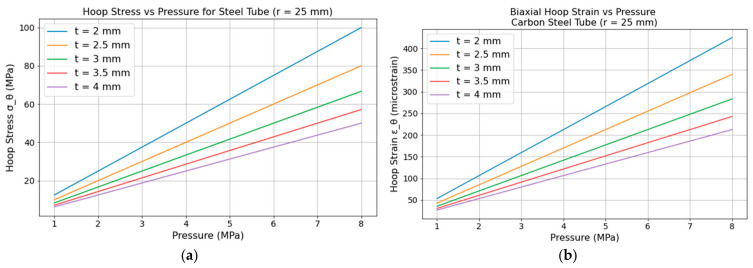
Analytical study in terms of varying pressure and fixed wall thickness: (**a**) hoop stress; (**b**) biaxial hoop strain.

The set of charts presented above provides a direct relationship between wall thickness and induced strain. These calculations are generally applicable to the outer surface of the straight section of a thin-walled tube. In the bend section, however, the stress distribution becomes more complex due to the curvature. The degree of this effect depends on the bend radius. Next, the effect of internal pressure on hoop stress and biaxial hoop strain for various wall thicknesses is examined. Figure 28 below shows a linear relationship, where thinner walls exhibit a steeper increase in stress and strain with rising pressure.

Mapping strain to FBG wavelength shift:

From the charts above, strain (Ɛ) can be estimated for a given thickness (t), radius (r), and internal pressure (P). Considering the strain sensitivity of an FBG sensor as (s) pm/µɛ (depending on packaging); the total strain-induced wavelength shift of an FBG sensor is expected to be (s. ɛ) nm. Considering the resolution of an FBG interrogator as (R) pm, i.e., the minimum detectable wavelength shift, the minimum measurable wall thickness change can be calculated as: t·Rs·Ɛ mm.

For example, for a 3 mm thick (t) carbon steel tube, with an outer radius of 25 mm (r), hoop strain (Ɛ) experienced at 7 MPa is 250 µɛ (from Figure 28b above). Typical FBG sensitivity (s) to strain is 1.2 pm/µɛ, and the resultant wavelength shift is as follows: 250 × 1.2 = 300 pm. Considering an interrogator with resolution (R) of 2 pm, the corresponding minimum detectable strain is as follows:

$$\frac{R}{s}=\frac{2}{1.2}\approx 1.67\;\mu \varepsilon$$

Using the analytical hyperbolic strain–thickness curve, the minimum wall-thickness change can be calculated as:

$$\frac{\Delta t}{t}=\frac{\Delta Ɛ}{Ɛ}=\frac{1}{150}$$

For t = 3 mm: $\Delta t\sim \;3\;\mathrm{mm}\;\times \;\frac{1}{150}=20\;\mu m$.

However, this linear extrapolation calculation is approximate and may not be directly applicable due to the nonlinear (hyperbolic) strain–thickness relationship. Experimental testing is therefore required to calibrate the sensing system, establish a clear mapping between wall thickness and strain (or FBG response), and define the tube failure threshold. Additionally, compensation for environmental variables is required to accurately determine absolute values of the induced mechanical strain from the FBG response.

- Applicability of FBG sensors in harsh environments, like tube boilers

Type II gratings are the structures inscribed within optical fiber using high-intensity laser pulses that exceed the damage threshold of the fiber core, resulting in permanent structural changes in the fiber core, which can be understood as the creation of micro-voids. Gratings produced in this way are highly thermally stable, i.e., they can withstand up to 800–1000 °C and are suitable for harsh environmental applications. Due to the permanent damage within the fiber core, their mechanical robustness is compromised. Study [212] investigating the performance of Type I and Type II through-coating FBG sensors highlights the thermal stability and fatigue trade-offs and suitability for defense applications. The authors in [213] presented research on the effectiveness of Type II gratings for temperature sensing in harsh environments, i.e., nuclear reactor environments.

**Figure 29:**
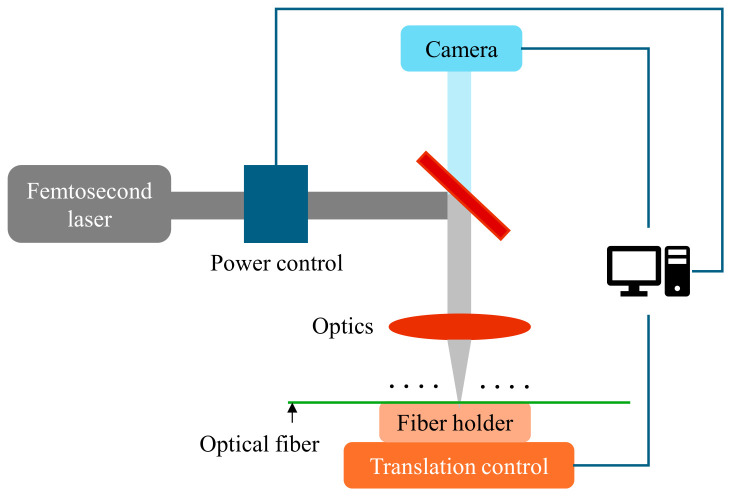
FBG micromachining set-up using a femtosecond laser (adapted from [214]).

The standard way of fabricating Type II gratings is by femtosecond laser (fs-laser) micromachining, or infrared (IR) irradiation, via point-by-point (PbP) or direct writing technique, as detailed in Figure 29. For such grating fabrication techniques, the underlying fiber does not need to be photosensitive as the high-intensity laser pulse interaction across tiny regions in a shorter timeframe produces high-resolution imprints [214]. The change in the refractive index in the FBG micromachining process occurs because of a nonlinear multiphoton process.

The optical fiber is placed on the fiber holder having a precise control of movement in three dimensions, X, Y and Z axes, using a translation controller. After the fiber alignment is achieved, the fs-laser is emitted from the Ti: sapphire laser source and focused directly onto the fiber. Each pulse creates a grating plane in the fiber, and the fiber is moved along its main axis at a translational speed (V) to write grating structure PbP. The period of grating now depends on the translational speed and repetition rate $\left(l_{fr}\right)$ of the laser, as in (10).

(10) Ʌ=Vlfr

Fabricating FBG sensors through micromachining is the primary step, followed by ensuring the fiber’s mechanical robustness so it can function reliably in harsh environments. Metal coatings, such as nickel, aluminum, copper or gold, are performed on standard silica fibers to enhance their robustness and durability, as well as their ability to survive at elevated temperatures and in harsh environments. The study reported in [215] details the suitability of nickel (Ni) coating on fibers due to its high melting point (1455 °C), citing electrodeposition as the most suitable deposition technique. Other techniques include physical vapor deposition (chemical or physical) and electroless chemical plating. Article [216] provides an understanding of multilayer metal-coated FBGs (nickel–copper–gold) for extremely high-temperature applications, e.g., 1000 °C. Metal coatings can also amplify sensor sensitivity due to differences in thermal expansion coefficients and provide a barrier against humidity and corrosive agents, enabling reliable performance in cryogenic and high-temperature applications, as reported in [217,218]. The authors in [219,220] demonstrated the performance of polyimide and gold-coated FBGs surface mounted on carbon steel tubes in temperatures ranging up to 500 °C. Gold-coated FBG sensors experience a remarkable temperature sensitivity of about 28 pm/°C.

An alternative approach involves fabricating FBG sensors within sapphire fibers, taking advantage of sapphire’s superior thermal and chemical stability compared to conventional silica fibers. Grating structures in sapphire fibers have attracted considerable attention for sensing applications in extreme environments, particularly under high-temperature conditions. Their main challenges lie in sensor fabrication (due to the multimode nature of sapphire fiber) and wavelength demodulation. Multiplexing of FBGs in a multimode sapphire fiber by fabricating ten cascaded gratings using femtosecond lasers is demonstrated in article [221], achieving strong reflectivity. The study confirmed temperature performance of the sensors in the range of 1500 °C. The authors in [222] proposed adding multimode fibers (AMMF) and tracking the longwave edge of the reflection spectrum to smoothen the mode–field distribution, improving stability. To address strain–temperature cross-sensitivity issues and facilitate their simultaneous detection, a dual-FBG configuration in sapphire fiber modified by wet acid etching is proposed in [223]. Experimental studies for temperatures ranging up to 1500 °C are reported.

### 4.3. Fiber Optic Sensors as a Forward-Looking Approach for Tube Bend Monitoring

With all the information presented above, here we propose fiber optic sensors as a forward-looking approach for tube bend monitoring, either as an alternative or as part of a hybrid solution complementing existing NDT methods. Research specifically focused on using fiber optic sensors to monitor tube bend wall thickness under boiler operational conditions remains largely unaddressed. We present a high-level system architecture for enabling tube bend condition monitoring with fiber optic sensors and identify key research directions to advance this technology toward practical deployment.

**Figure 30:**
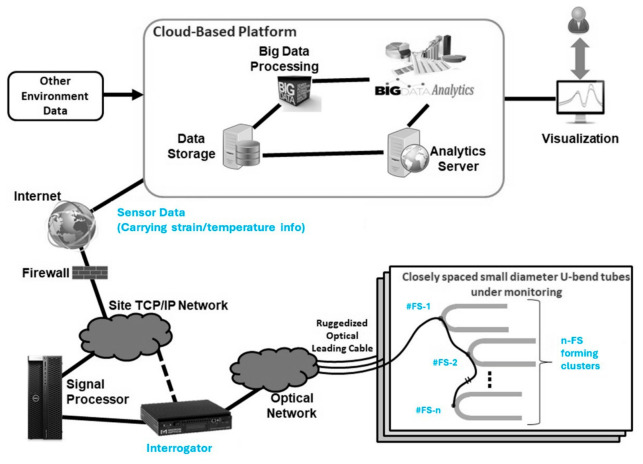
System architecture: fiber optic sensing solution for tube bend monitoring.

A system architecture of fiber optic sensing solutions as a forward-looking approach for tube bend monitoring is shown in Figure 30. Points #FS-1/2/…n represent the fiber sensor’s discrete points of installation (in the case of FBG) or spatial sampling points (in the case of DFOS), forming a cluster of sensors along the optical fiber. Sensors are expected to be installed at critical locations on the outer wall of tube bends. These tubes are generally closely spaced, small-diameter tube bends operating in harsh environments. Critical locations on the tube are selected based on their damage probability, i.e., bend/turn region. Sensor mounting approaches such as epoxy bonding and spot welding are critical for ensuring reliable strain transfer while accommodating quasi-distributed architectures. Epoxy adhesion, often using high-temperature ceramics, provides a robust, conformal attachment to curved tube surfaces, minimizing slippage and enabling uniform strain distribution across multiplexed gratings; however, it demands precise surface preparation to avoid microbubbles in corrosive settings. Spot welding, typically applied to weldable shims or H-shaped mounts pre-attached to the sensing fiber, offers a vibration-resistant alternative suitable for harsh environments, as demonstrated in strain gauge-like deployments with thermal expansion matching structural material. Hybrid methods combining epoxy for fiber encapsulation with spot-welded substrates enhance survivability and early defect sensitivity by decoupling thermal effects, but packaging challenges persist in scaling for dense placement.

Referring to Figure 30 above, fiber sensor signals are coupled to an optical network via ruggedized optical leading cables, which are then configured to an interrogator. The interrogator is provided to read and capture the sensor data in real time. The sensor data can be coupled to a computing platform (for example, a cloud-based platform) via a communication network (TCP/IP, internet). The platform comprises data storage modules, analytic servers and data processing modules to process and analyze the raw data to get an output, which may be connected to a display device for visualization. The necessary environmental data—e.g., temperature, pressure, flow rate at various locations of feed water, evaporator tank, boiler inlet and outlet, etc.—may also be provided to the computing platform to deduce the absolute strain profile after completing feature engineering.

This review identifies critical research directions highlighted below to advance fiber-based solutions for tube bend monitoring, including robust sensor packaging, high-temperature performance validation, strain–temperature compensation algorithms, big-data analytics for predictive insights, and scaled-down failure demonstration and validation lab tests.

(1) Sensor packaging: Optimized packaging remains essential to secure optical fiber on curved tube surfaces under dynamic/vibrational loads, preventing delamination or slippage while maintaining signal integrity. Research must explore advanced adhesives, composite embeddings, and miniaturized housings compatible with corrosive coatings, addressing current limitations in long-term adhesion and multiplexing scalability [224,225]. Performance of surface mounted FBG sensors on carbon-steel tube bends at up to 500 °C is demonstrated using polyimide-coated fiber [219] and gold-coated fiber [220].
(2) High-temperature performance and compensation algorithms: Multiple rounds of test characterizations of fiber sensors mounted onto industrial tubes operating at high-temperature are required to quantify thermal crosstalk, sensitivity, accuracy, wavelength stability, etc. Algorithms to decouple strain from temperature variations are vital, leveraging reference FBGs or machine learning to enhance accuracy in fluctuating environments and to improve early defect detection.
(3) Big data processing of sensor signals: Research is required to develop scalable edge or cloud analytics for defect detection, pattern analysis, segmentation, strain gradients, and predictive failure modeling. Research into federated learning and reduced-latency processing will enable actionable insights from continuous monitoring, bridging raw spectral data to operational decisions. The authors in [226] presented a data-driven framework for real-time monitoring of tube wall thinning in dynamic noisy conditions, having the fiber sensors installed on the small-diameter, closely spaced tube bends in a real boiler environment, with a vast amount of spatial–temporal data collected and classified into thickness-related features, enabling a statistical monitoring scheme for thinning or abnormality detection. Problem formulation incorporating local or global environmental variables, e.g., pressure or temperature, etc., along with fiber sensor data is explained, forming the basis of such data-driven model development.
(4) Scale-down failure test: Research or demonstration experiments in a controlled environment simulating tube bend failures at laboratory scale are required to validate and confirm sensors’ applicability in such applications, studying wavelength mappings, benchmarking, thresholding, and reliability checks.

Next, Table 9 provides a summary of the qualitative and quantitative characteristics of fiber optic sensors, along with their operability in such application environments.

## 5. Recommendations

Conventional NDT techniques, though widely employed in power and process industries, face significant limitations when applied to boiler tube bends. These curved sections of tubing are critical components that frequently experience severe thermal, mechanical, and chemical stresses during operation. Unlike straight tube sections, bends are more susceptible to localized degradation, such as wall thinning due to accelerated corrosion–erosion, fatigue cracking from cyclic stresses, or even stress corrosion cracking under high-temperature and high-pressure environments.

A primary challenge arises from the geometric complexity of tube bends. Their curvature alters stress distribution and flow dynamics of steam, making them more prone to localized damage compared to straight segments. This geometry complicates the deployment of many conventional NDT methods, such as ultrasonic testing, eddy current testing, or radiography, that typically assume relatively uniform geometrical profiles to ensure accurate measurement. Technicians often struggle to position probes, transducers, or visual inspection equipment precisely on confined space and bends. This challenge greatly reduces detection reliability, especially for small-scale defects or early stages of wall thinning.

The environmental conditions within boilers present yet another major obstacle. Tube surfaces exposed to high temperatures, internal pressure, slag deposition, and combustion byproducts create a harsh background for inspection. These factors together diminish the applicability of traditional NDT techniques and require partial/complete shutdown of equipment to conduct meaningful inspections, translating into a direct revenue loss.

Another recognized shortcoming of conventional NDT methods lies in their operational requirements. Inspections are generally conducted manually by skilled workers and typically require trained personnel for results interpretation. More importantly, manual inspections provide only periodic snapshots of the tube’s health. They cannot offer continuous or real-time monitoring, meaning that the condition of boiler tube bends between scheduled inspections remains unknown. Early damage progression may therefore go undetected until it reaches critical severity.

In harsh operating environments, such undetected degradation raises the risk of sudden tube rupturing, which can result not only in costly unplanned outages but also in serious safety hazards for operators. Consequently, the timely identification of localized damage in tube bends remains one of the pressing inspection challenges faced by industries.

- Fiber optic sensing as an alternative or hybrid solution

In response to the limitations of conventional approaches, fiber optic sensing technologies could offer a groundbreaking alternative for boiler tube inspection and monitoring. Among these, femtosecond laser micromachined FBG sensors are well suited due to their ability to deliver real-time, in-service condition assessments under harsh environment. Unlike traditional methods, fiber optic sensing systems can operate while the plant remains online, eliminating the need for costly shutdowns. These systems can provide continuous measurements with high sampling rates, thereby enabling the early detection of degradation in critical components such as tube bends, preventing catastrophic failures.

FBGs are intrinsically immune to electromagnetic interference, allowing them to perform reliably in the electrically noisy environments typical at power industries. Secondly, their miniature size and flexible deployment make them particularly suited for surface attachment at sensitive or critical locations. Thirdly, fs-laser micromachined FBGs can endure harsh operating environments, including elevated temperatures, thermal cycling, and chemically reactive atmospheres. This durability of grating makes them highly preferable for long-term monitoring of boiler tubes under real operating conditions.

These femtosecond laser micromachined FBG sensors are capable of providing quasi-distributed sensing solutions by deploying multiple gratings along a single optical fiber, covering the distinct points along a tube. Such multiplexing capability offers a comprehensive health map of the component. In practice, integration of fiber optic sensing systems with early-warning modules allows operators to implement maintenance interventions before failures escalate into disasters, thereby reducing risks, operational costs, and unplanned outages, advancing next-gen digital plant management systems.

Despite these promising advantages, it is important to acknowledge that current research on deploying these micromachined FBG sensors directly for boiler tube bend integrity monitoring remains relatively limited or negligible. The concept therefore remains underexplored, presenting a fertile research direction for scientists, engineers, and industrial practitioners. Systematic investigations into sensor design, packaging methodology, deployment techniques, characterization and calibration under realistic operating conditions, and integration with plant monitoring infrastructure, will be essential to fully realize the potential of this technology and its strong recommendation.

Prospective studies may focus on hybrid monitoring frameworks that combine fiber optic sensing with advanced data analytics, machine learning, and predictive modeling techniques. These integrated approaches could unlock unprecedented levels of reliability and efficiency in boiler tube integrity management. As such, fiber optic sensing stands as a transformative approach that could redefine the landscape of non-destructive evaluation in the power generation sector.

## Figures and Tables

**Table 1:** Key parameters across specified standards (data from [32]).

Std.	Material Type	OD (mm)	WT (mm)	Temperature Range (°C)	Pressure Range	Use Cases
A179	Low-carbon steel	12.7–76.2	1–9	<350	Low	Heat exchangers, condensers
A192	Carbon steel	12.7–177.8	2.2–25.4	<450	High	High-pressure services
A209	Carbon–molybdenum	12.7–127	2–12	<500	Medium–High	Boilers, superheaters
A210	Medium-carbon steel	12.7–114.3	0.8–15	<450	Medium	Boilers, superheaters
A213	Ferritic and austenitic alloy	6–1240	1–50	<650	High	Superheaters, heat exchangers
A335	Ferritic alloy	6–1240	1–50	<700	High	Power plants, refineries
DIN 17175	Carbon/alloy	10–762	1–80	<600	Medium–High	Boilers, pressure vessels
JIS G3461	Carbon steel	15.9–139.8	1.2–12.5	<350	Low–Medium	Boilers, heat exchangers
JIS G3462	Alloy steel	15.9–139.8	1.2–12.5	<600	High	High-pressure boilers

**Table 2:** Chemical composition across specified standards (data from [32]).

Std.	C (%)	Mn (%)	P (%)	S (%)	Si (%)	Cr (%)	Mo (%)
A179	0.06–0.18	0.27–0.63	<0.035	<0.035	<0.25	-	-
A192	<0.25	0.27–0.63	<0.035	<0.035	<0.25	-	-
A209 T1	0.10–0.20	0.30–0.80	<0.025	<0.025	0.10–0.50	-	0.44–0.65
A210 A1	<0.27	<0.93	<0.035	<0.035	>0.10	-	-
A213 T11	0.05–0.15	0.30–0.60	<0.025	<0.025	0.50–1.00	1.00–1.50	0.44–0.65
A335 P11	0.05–0.15	0.30–0.60	<0.025	<0.025	0.50–1.00	1.00–1.50	0.44–0.65
DIN 17175	<0.17	0.40–0.80	<0.040	<0.040	0.10–0.35	-	-
JIS G3461	<0.18	0.30–0.60	<0.035	<0.035	0.10–0.35	-	-
JIS G3462	0.05–0.15	0.30–0.60	<0.035	<0.035	<0.50	0.80–1.25	0.45–0.65

**Table 3:** Mechanical properties across specified standards (data from [32]).

Std.	Yield Strength (MPa)	Tensile Strength (MPa)	Elongation (%)	Hardness (HRB/HB)
A179	>180	>325	>35	<72 HRB
A192	>180	>325	>35	<77 HRB
A209 T1	>205	380–550	>30	<80 HRB
A210 A1	>255	>415	>30	<79 HRB
A213 T11	>205	>415	>30	<85 HRB
A335 P11	>205	>415	>20	<85 HRB
DIN 17175	>235	360–480	>25	<75 HRB
JIS G3461	>175	>340	>35	<77 HRB
JIS G3462	>205	>410	>30	<85 HRB

**Table 4:** Summarizing key attributes of existing NDT techniques.

Method	Key Advantages	Key Limitations	Typical Sensitivity	Typical Penetration Depth
RVI	Simple, fast, low cost; good for obvious surface flaws, corrosion, misalignment; remote tools (borescopes, drones) access confined areas	Only surface-visible defects; strongly dependent on lighting, accessibility, and inspector skill; no volumetric information	Good for medium–large surface defects (cracks, pits, corrosion, lack of alignment); poor for very tight or subsurface flaws	Surface only; no true subsurface penetration
UT	Detects internal flaws; good depth reach; can size defects; suitable for thickness measurement; fast and portable	Needs good surface contact and couplant; interpretation requires skilled operators; geometry and coarse grains can reduce reliability	High for planar defects like cracks and lack of fusion; can detect small defects relative to thickness	Up to several hundred millimeters in metals (depending on material and frequency
RT	Direct image of internal volumetric defects (porosity, inclusions); permanent record; relatively insensitive to surface condition	Radiation hazards and strict safety controls; relatively slow and costly; less sensitive to tight planar cracks; often needs access to both sides	High for volumetric defects; moderate for tight planar defects	Good penetration in thick and dense sections (tens to hundreds of millimeters depending on energy and material)
ECT	Non-contact and fast; very sensitive to small surface and near-surface defects; also measures conductivity, coating thickness, and material changes	Only for conductive materials; limited penetration; results affected by lift-off and geometry; requires calibration and skilled interpretation	Very high for small surface-breaking cracks and near-surface defects	Typically, a few millimeters at most, depending on frequency and material
EMAT	Non-contact generation and reception of ultrasound; tolerant of rough or scaled surfaces; suitable for high-temperature components; easy automation and scanning	Requires conductive or ferromagnetic materials; lower signal amplitude than conventional UT, so sensitivity and penetration may be reduced; high equipment cost	Moderate to high for certain defect orientations; generally lower SNR than contact UT, so very small flaws can be harder to detect	Similar order of magnitude as UT but often somewhat reduced because of lower signal strength
IRT	Full-field, fast imaging over large areas; non-contact; and suitable for detecting disbonds, delamination, and surface heat anomalies; useful for in-service monitoring	Primarily surface and near-surface; requires controlled excitation and emissivity correction for quantitative work; limited defect sizing in depth; affected by environmental conditions	High for near-surface defects that significantly disturb the thermal field (disbonds, shallow cracks, inclusions)	Typically, up to a few millimeters in metals and several millimeters to centimeters in low-conductivity materials (e.g., composites), depending on excitation

**Table 5:** Applicability with respect to high temperatures and complex tube bend geometry.

Method	High-Temperature Applicability	Applicability to Complex Tube Bend Geometries	Thickness Monitoring (Qualitative OR Quantitative)
RVI	Suitable for hot components with protected optics and line-of-sight access; widely used for in-service hot piping and boiler inspections [135]	Applicable if bends are accessible (e.g., borescopes/crawlers/fiberscopes); geometry affects navigation [135]	Qualitative via visual estimation
UT	Require couplants; works up to 200–350 °C with PAUT probes; compensating for velocity/attenuation changes [135,136]	Limited by probe size; challenging due to varying angles and surface curvature; automated orbital scanners around bends are commonly used [135,136]	Quantitative via thickness mapping
RT	Temperature minimally affects imaging via X-ray/gamma source scan, though access/safety limits apply [137]	Challenging to bends due to source-detector positioning, difficult image interpretation due to varying thickness through the bend radius [137]	Quantitative via density profile comparisons knowing the material properties in advance
ECT	Relatively temperature-insensitive vs. UT; require probe insulation viable up to ~300 °C with insulated probes [135,138]	Widely used for boiler inspections, though tight radii reduce coupling uniformity and complicate sizing [136]	Quantitative via amplitude and phase analysis
EMAT	Non-contact EMATs enable ultrasonic testing at high temperatures with reduced signal amplitude [139]	Complex but theoretically possible using dedicated shoes or robotic carriers [140]	Quantitative-like UT thickness mapping
IRT	Suitable for high-temperature components as it detects emitted radiation [135]	Challenging due to geometry-induced non-uniform heating and view factors [135]	Qualitative interpretation via thermal patterns only

**Table 6:** Latest case studies on wall thickness reduction detection.

Case Studies (Paper)	Error (Resolution)	Sensitivity (SNR)	Penetration Depth (Range)	Speed	Robustness
SH Ultrasonic Waveguide (2019, FAC facility, carbon steel pipe 5.54 mm nominal) [77]	±15–20 μm	High SNR via pitch/catch technique	Local contact (point measurement)	Real-time (hourly)	High temp (up to 150 °C, 3300 h stable); flow velocity (7–12 m/s)
EMAT Fuzzy Logic Fusion (2024, metals 0.3–1000 mm) [141]	±1.2 mm (max error)	SNR improved by Linear Frequency Modulation	0.3–1000 mm range (accuracy: 1%)	Sweep-based (secondary measurement)	Non-contact; broad range; temp stable (room)
Laser Ultrasonic PSO-VMD (2023, ductile iron pipes 4.2/6.2 mm) [142]	~96% (accuracy)	SNR from 35 to 43 dB	Thin walls with inclusions (4–6 mm)	Scanning (B-scan images)	Detects slag defects; denoised signals
Guided Wave Frequency-Wavenumber (2022, steel pipes) [143]	<1.5% (error)	-	Circumferential scanning (mean thickness)	Multi-sensor (12–72 sensors)	Noise robust; uniform thinning (less for local dents)
EMAT Linear Array Reflected Modes (2017, steel pipe 10.2 mm) [144]	Max 0.4 mm (error)	-	600 mm propagation	Guided wave (remote)	Defect localization ±10 mm; non-contact

**Table 7:** Summary: Fiber optic solutions to conventional NDT constraints.

Conventional NDT Limitation	Fiber Optic Sensing Solution
Contact requirements (UT needs couplant; ECT needs proximity)	Non-contact after installation; surface-mounted, attached, or welded
Point measurements only (discrete probe locations; periodic operation)	Possible to have distributed or quasi-distributed sensing fiber providing continuous strain profiles [145]
High-temperature limits (UT/ECT < 350 °C; RVI optics may fail or underperform)	Intrinsic high-temperature operation due to silica glass fiber; and no electronics involved at sensing point [146]
Poor complex geometry access (tube bends, occluded areas)	Conformable sensors: fibers follow exact pipe contours, welded joints, bend radii; multiplex hundreds of FBGs or distributed points in single cable [147]
Manual inspection only (shutdowns required)	Continuous online monitoring: permanent sensors detect defect progression according to desired intervals [148]
Radiation hazards and access (RT logistics)	No radiation, no access needed post-installation; single interrogator serves entire network
Surface condition dependency (coatings, scale defeat UT/ECT)	Immune to surface roughness, scale, paint—measures internal strain distribution and defects [149]

**Table 8:** Case studies of fiber optic sensors applied in pipeline monitoring.

Ref.	Underlying Technology	Target Application (Parameter)	Evaluation Environment	Performance Parameter
[164]	DFOS	Gas pipeline leakage	Laboratory test with standard industrial pipe	-
[165]	DFOS (DSS)	Pipeline’s integrity via strain	Laboratory test with high-density polyethylene pipe	-
[167]	DFOS (DSS)	Pipeline’s bend and dent	Laboratory test with steel pipes	-
[168]	DFOS (phase-OTDR)	Gas pipeline’s vibration	Field trial on natural gas pipeline	-
[169]	DFOS (DTS)	Pipeline leakage via temperature	Scale-down laboratory test	-
[170]	DFOS (DSS)	Pipe–soil interaction via strain	Model test with analytical model	-
[171]	DFOS (DSS)	Pipe corrosion via strain	Laboratory test with steel pipes	-
[172]	FBG	Pipeline corrosion via strain	Laboratory test with pressurized steel pipes	Sens.: 136.32 pm/mm^−1^
[173]	DFOS (DSS)	Corrosion via strain	Laboratory test with steel bar	-
[174]	DFOS (DSS)	Corrosion via strain	Laboratory test with marine steel piles	Sens. of mass loss: 0.00005 g/h, 0.00403 g/h, 0.00635 g/h, and 0.00454 g/h in the atmospheric, water level fluctuation, immersion, and sub-mud zone
[175]	DFOS (DAS)	Pipeline intrusion	Field trial	-
[176]	DFOS (DAS)	Pipeline blockage/clogging	Laboratory test	Strong acoustic signals in front of the clogging point
[177]	Point/multi-point	Pipeline leakage and corrosion via strain	Laboratory test with PVC pipes	Sens.: 1767 με/mm (for PVC pipe)
[178]	Point/multi-point	Liquid level monitoring via strain	Laboratory test	Sens.: 27 pm/cm Resolution: 0.04 cm Error: 0.1%
[179]	Point/multi-point	Wastewater effluent via refractive index	Laboratory test	Sens.: 0.76 pm/ppm (Cl) 38.6 pm/ppm (Pb) Detection limit: 1.2 ppm (Cl) 0.003 ppm (Pb)
[180]	Point/multi-point	Liquid level monitoring via strain	Field trial in industrial water tank	Sens.: 2.8 pm/mm
[181]	Point/multi-point	Liquid pressure sensor via strain	Laboratory test	Sens.: 18.651 nm/MPa Range: 0.36 MPa
[182]	Point/multi-point	Water level sensor via strain	Laboratory test	Sens. 155.7 ± 2.4 pm/m
[183]	Point/multi-point	Temp and pressure in oil and well application	Laboratory test	Pressure sens.: 24.05 pm/MPa Range: 40 MPa Temp. sens.: 31.16 pm/°C Range: 200 °C
[184]	Point/multi-point	Petroleum hydrocarbon leak detection via strain	Laboratory test	2 nm under 20 min gasoline influence
[185]	Point/multi-point	Temperature measurement in synchrotron radiation source	Field trial at Indus-2 operation	Accuracy: ±1 °C
[186]	Point/multi-point	Gas detection (acetylene)	Laboratory test	Matching 20 nm region of p-band absorption spectrum of acetylene
[187]	Point/multi-point	Gas detection (carbon dioxide)	Laboratory test	Sens.: 3.39 pm/mM; Detection limit: 0.087 mM
[188]	Point/multi-point	Moisture content detection of sandstone via strain	Laboratory test	Sens.: 10,240 pm/(g∙g^−1^) Detection limit: 0.01 (g∙g^−1^)
[189]	Point/multi-point	Boiler hanger rod via strain	Field trial in industrial boiler	-
[190]	Point/multi-point	Thermal cycling of steel beam	Laboratory test	Error: <2%
[191]	Point/multi-point	Optical fiber thermometer	Field trial	Range: 1200 °C

**Table 9:** Performance characteristics of fiber optic sensors in application environments.

Aspect	FBG-Based (Point/Quasi-Distributed)	DFOS (Brillouin/Rayleigh)-Based
Sensing principle	Bragg wavelength shifts due to axial strain and temperature at discrete grating locations, often arranged at different points around circumference [227,228]	Continuous measurement of Brillouin or Rayleigh frequency shift along fiber, giving distributed axial or hoop strain and temperature; bend behavior inferred from strain field along a tube [229,230]
Configuration on tube bends	Small arrays (e.g., 4–32 FBGs) clustered around bend; gratings multiplexed along a single fiber (fixed spacing over the bend) for quasi-distributed strain mapping [228,231]	Fiber bonded or clamped along axial, spiral, or hoop paths following the bend; single cable can cover many sections with one interrogator channel [230,232]
Spatial resolution	Point sensing; effective gauge length ≈ grating length (typically 3–10 mm), with sensor spacing defined by multiplexing design (few mm to cm)	Set by DFOS underlying technology, high-resolution setups down to 10 cm demonstrated for wall-thinning defects [229]
Sensitivity	Four-core FBG bend sensor: curvature resolution ≈ $3.6\times 10^{-3}$ m^−1^ at 1.5 µm [231]; typical strain sensitivity ≈ 1.2 pm/µε	Strain resolution typically 10–20 µε; measurement accuracy better than 10 µε and 0.5 °C; sufficient to resolve mm-scale wall-thickness changes via hoop strain
Dynamic response	Sampling at kHz-range possible	Typical DFOS systems operate from tens of seconds per full scan; quasi-static assessment but not for high-frequency vibration, except DAS
Measurement range	Wavelength-encoded; can track strains to several thousand µε before nonlinearity or fiber yield	Long-range fiber coverage (km); strain meas. range depends on length of fiber under test
Temperature performance	FBGs operate to 300–400 °C with suitable coatings; regenerated or specialty gratings and sensors can survive harsh environment in steam or furnace environments when properly packaged [219,220,227]	Standard DFOS cables rated up to ≈80–120 °C; high-temperature DFOS cables require special packaging, but industrial implementations on very hot boiler bends remain less mature
Cross-sensitivities	Intrinsic strain–temperature cross-sensitivity exists, requiring reference FBGs or algorithmic compensation	Brillouin frequency shift depends on both strain and temperature; need independent temperature compensation or dual-path configurations
Installation on bends	Requires robust bonding on curved surfaces, often with grooves, stainless-steel carriers, or multi-core rods to maintain strain transfer and survivability	Armored fiber cables bonded along the pipe; for bends, hoop placement must manage minimum bend radius; installation is less geometrically constrained than discrete FBG packages
Survivability in harsh environment	Bare silica is chemically robust but requires protective jackets, coatings, and metallic packaging near boiler bends; long-term performance limited by coating degradation, pitting-induced hoop strain, and mechanical damage during operation	Cable jackets and armoring provide good mechanical protection; long-term operation demonstrated on submerged pipelines, though direct exposure to highly aggressive boiler environments is less researched
Interrogator complexity and cost	FBG interrogators with sub-pm resolution; cost scales with channel count and speed, but channel-sharing allows many points per unit	DFOS interrogators are more complex and costly per unit but cover tens of km; cost per meter of monitored pipe can be favorable for large infrastructure
Suitability for real-time monitoring	Highly suited for real-time online monitoring of critical bends, including early defect prediction	Ideal for quasi-static monitoring, periodic wall-thinning progression assessment

## Data Availability

No new data were created or analyzed in this study. Data sharing is not applicable to this article.
